# Obtaining single crystals containing cationic porphyrins from aqueous solutions: a systematic approach including nano-crystallization, organic modifiers and gel crystallization

**DOI:** 10.1107/S2052252525009716

**Published:** 2026-01-01

**Authors:** Rayk A. Schmitz, Florian C. Brunner, Leonard P. Zelder, Bernhard Spingler

**Affiliations:** a Department of Chemistry University of Zurich Winterthurerstrasse 190 Zürich 8057 Switzerland; Sun Yat-Sen University, China

**Keywords:** porphyrins, gel crystallization, robotic pipetting, crystallization screens, crystal growth, X-ray crystallography

## Abstract

Obtaining high-quality single crystals containing charged molecules from aqueous solutions can sometimes be challenging. In this publication, we studied the crystallization of polycationic porphyrins. Crystals of three polycationic porphyrins, [5,10,15,20-tetrakis(1-methylpyridin-1-ium-4-yl)porphyrin, 5-(4-cyanophenyl)-10,15,20-tris(1-methylpyridin-1-ium-4-yl)porphyrin and 5-(4-carboxy­phenyl)-10,15,20-tris(1-methylpyridin-1-ium-4-yl)porphyrin], were obtained with different anions. We started initially with ‘nano-crystallization’ screening in order to identify promising combinations of the cations with suitable anions, followed by optimizing the ratio of anions to cations. Both steps yielded crystals, leading to the determination of one porphyrin-containing crystal structure from each step. In the next step, five different organic solvents were trialled as additives in an aqueous hanging-drop crystallization setup, which led to the determination of three more crystal structures. It was shown that it was possible to improve the quality of the crystals without the organic additive being incorporated into the crystal structure. Finally, crystals of one porphyrin salt were obtained by gel crystallization. These crystals were higher quality than the crystal grown by vapour diffusion, leading to a high-quality and higher-resolution X-ray structure determination. This is the first porphyrin structure to be determined from a crystal grown by gel crystallizaton.

## Abstract 2

A stepwise guide to the crystallization of charged molecules from aqueous solutions is presented.

## 1. Introduction

Our group has developed an aqueous, extremely powerful high-throughput screening (HTS) method, called nano-crystallization, in order to efficiently identify combinations of an organic cation with various organic or inorganic anions that lead to the formation of crystals. The nano-crystallization method can be performed either as a vapour-diffusion technique (Nievergelt *et al.*, 2018 ▸; Alvarez *et al.*, 2020 ▸) or as an under-oil technique (Babor *et al.*, 2019 ▸). As described in these publications, this method allows the crystallization behaviour of a cation with 96 different anion solutions, containing 77 different anions, to be tested simultaneously. The choice of the anions was based on good water solubility, known ability to form crystals [*e.g.* tetra­fluoro­borate (Kurz *et al.*, 2005 ▸), tetra­phenyl­borate (Kuzelka *et al.*, 2002 ▸; Medina-Molner *et al.*, 2015 ▸) or hexa­fluoro­phosphate (Baumeister *et al.*, 2002 ▸; Hernández-Valdés *et al.*, 2020 ▸)], safety concerns (therefore azide and perchlorate were not chosen), commercial availability (at least the corresponding acid of the Brønsted bases should be commercially available) and different basicities (p*K*_b_ values). The vapour-diffusion variant of this method only needs 100–500 nl of the analyte stock solution per crystallization experiment, potentially yielding high-quality single crystals which can subsequently be analysed directly using a home microsource single-crystal X-ray diffractometer. The cation stock solutions are pipetted into the corresponding wells by a pipetting robot, saving valuable time for the scientist.

**Figure 1:**
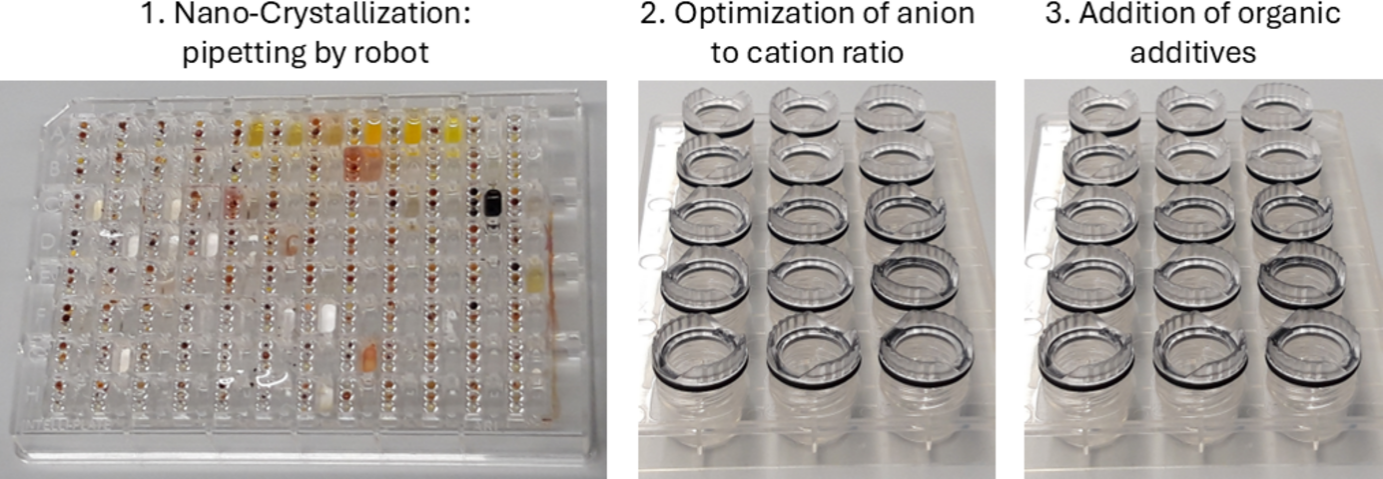
Optimization steps if the initial nano-crystallization screen does not yield a sufficiently large crystal of the required quality.

However, in some cases, the crystals that are obtained are too small, and optimization of the initial hits must be performed. In the current work, three methods (see Fig. 1 ▸) were employed to improve the quality and increase the size of the crystals that were initially obtained:

(1) reduction of the molar excess of the anion versus the cation,

(2) addition of organic neutral additives, or

(3) gel crystallization.

While the first and the second method were mentioned in individual cases in the supporting information of our original publication on nano-crystallization (Nievergelt *et al.*, 2018 ▸), in the present work these methods are tested with a systematic approach.

The nano-crystallization method (Nievergelt *et al.*, 2018 ▸; Babor *et al.*, 2019 ▸; Alvarez *et al.*, 2020 ▸; Rubbiani *et al.*, 2020 ▸) employs a molar excess of the anion versus the cation to be crystallized. If crystals of sufficient quality result, this is a very efficient way of crystallizing a substance, because the stoichiometries of anion and cation do not need to be adjusted. On the other hand, lowering the excess amount of the anion might allow the crystal quality to be improved in a very simple way.

As an alternative way to improve the quality of the porphyrin crystals obtained in the HTS within the 96-well plates that are used for nano-crystallization, the addition of neutral organic co-solvents to the aqueous crystallization setup can be considered. Previous research has shown that organic additives can lead to improved X-ray diffraction and extend the diffraction limit. These advantages have been reported in particular for the crystallization of macromolecules like zeolites (Sun & Shen, 2012 ▸), DNA (Spingler *et al.*, 2001 ▸; Rohner *et al.*, 2016 ▸) and proteins (Luberacki *et al.*, 2008 ▸; Gosavi *et al.*, 2009 ▸; McPherson *et al.*, 2011 ▸). The effect of intentionally included organic additives on the aqueous crystallization of inorganic salts has been studied (Paulaime *et al.*, 2003 ▸; Nahi *et al.*, 2021 ▸). The shape of urea crystals can be influenced by addition of the neutral molecule biuret to the aqueous crystallization solution (Salvalaglio *et al.*, 2012 ▸). Most recently, the impact of 1 mol% of added amino acids was studied during the crystallization of α-glycine (Offiler *et al.*, 2025 ▸). However, to the best of our knowledge, our work from 2018 (Nievergelt *et al.*, 2018 ▸) remains one of the very few reports about improving the crystallization of small molecules from aqueous solution with the help of organic additives for the purpose of single-crystal structure analysis.

Gel crystallization is a method which can be used on almost every system with respect to solvents and analytes to be crystallized. Crystals obtained by gel crystallization can have improved properties, such as quality, size, shape and fewer defects, compared with those obtained using other techniques. Gel crystallization can be used with aqueous and non-aqueous solvents (Choquesillo-Lazarte & García-Ruiz, 2011 ▸) and done in either counter-diffusion (Artusio *et al.*, 2021 ▸), vapour-diffusion (Choquesillo-Lazarte & García-Ruiz, 2011 ▸) or layering (Yaghi *et al.*, 1997 ▸; Rizzato *et al.*, 2016 ▸) mode. When the latter approach is used, the layering of different ion solutions becomes very simple, reducing mixing of solutions and saving time in the laboratory. Because virtually no solvent evaporation takes place, the sedimentation of ions is reduced to a minimum, which results in fewer impurities in the crystals (Henisch, 2005 ▸).

Porphyrins are a class of organic compounds characterized by their macrocyclic structure, composed of four pyrrole subunits linked by methine bridges to form a conjugated aromatic system. The growth of high-quality porphyrin crystals is crucial for obtaining structural information (Tsalu *et al.*, 2015 ▸; Kingsbury & Senge, 2021 ▸) about the vast diversity of porphyrins, which are present in all living organisms, used as photosensitizers for collecting solar energy (Gu *et al.*, 2022 ▸; Mehrzad Sajjadinezhad *et al.*, 2024 ▸) and have clinical (Maharjan *et al.*, 2022 ▸) as well as potential oncological applications (Lam *et al.*, 2019 ▸; Xu *et al.*, 2023 ▸; Donohoe *et al.*, 2023 ▸; Gao *et al.*, 2024 ▸). On the other hand, growing high-quality crystals of porphyrins with sufficient size is a challenge (Lee *et al.*, 2008 ▸; Medforth *et al.*, 2016 ▸). The tetracationic [5,10,15,20-tetrakis(1-methylpyridin-1-ium-4-yl)porphyrin]^4+^ ([TMPyP]^4+^), which is a powerful telomerase inhibitor (Wheelhouse *et al.*, 1998 ▸), is being evaluated as an anti-bacterial photosensitizer (Malara *et al.*, 2017 ▸) and damages DNA by singlet oxygen generation (Tada-Oikawa *et al.*, 2009 ▸). Scheidt *et al.* (2015 ▸) have crystallized 5,10,15,20-tetrakis(1-methylpyridin-1-ium-4-yl)porphyrin 5,10,15,20-tetrakis(4-sulfonatophenyl)porphyrin with the help of a U-tube by counter-diffusion in polypropyl­ene glycol (PPG). These authors also mentioned that they were unable to crystallize their molecules of interest with the help of a gel. We have previously shown that the tricationic [5-(4-cyano­phenyl)-10,15,20-tris(1-methylpyridin-1-ium-4-yl)porphyrin]^3+^ ([TriMCNP]^3+^) is an efficient photodynamic therapy (PDT) photosensitizer upon irradiation with light of a wavelength greater than 600 nm (Antoni *et al.*, 2015 ▸). Finally, tricationic [5-(4-carboxyphenyl)-10,15,20-tris(1-methylpyridin-1-ium-4-yl)­porphyrin]^3+^ ([TriMCOOP]^3+^) has been tested as a telomerase inhibitor (Shi *et al.*, 2001 ▸) and used multiple times as a part of porphyrin conjugates (Mion *et al.*, 2015 ▸; Spagnul *et al.*, 2017 ▸; Tosto *et al.*, 2024 ▸).

**Figure 2:**
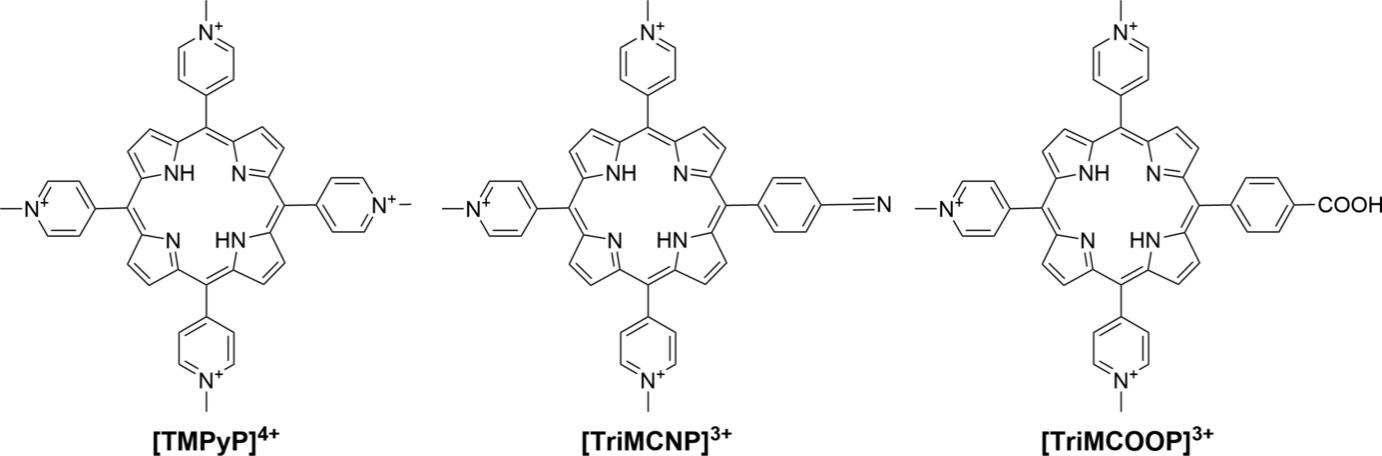
The polycationic porphyrins studied.

To the best of our knowledge, no porphyrins have been crystallized with the help of gel crystallization. Since the chosen cationic porphyrins, [TMPyP]Cl_4_, [TriMCNP](NO_3_)_3_ and [TriMCOOP]Cl_3_ (Fig. 2 ▸) are all soluble in water above the lower limit of concentration, which we previously found to be important (2 mg ml^−1^; Nievergelt *et al.*, 2018 ▸), ‘normal’ aqueous crystallization was expected to be possible either as vapour diffusion or gel crystallization with either agarose (Moreno & Rosales-Hoz, 2017 ▸) or tetra­meth­oxy­silane (TMOS) (Kumar & Steed, 2014 ▸; Rizzato *et al.*, 2016 ▸; Sumida *et al.*, 2017 ▸; Sánchez-Vergara *et al.*, 2017 ▸). Sometimes silicate gels are used for gel crystallization, which are prepared from sodium silicate (‘water glass’) followed by acidification (García-Ruiz *et al.*, 1998 ▸). We decided not to use these silicate gels, as they intrinsically contain an additional salt and therefore might interfere with the intended anion exchange of the original nano-crystallization process.

## 2. Results and discussion

The three different techniques used to crystallize the tetracationic [TMPyP]^4+^ and the tricationic [TriMCNP]^3+^ and [TriMCOOP]^3+^ porphyrins (Fig. 2 ▸) were vapour diffusion with either sitting- or hanging-drop crystallization, and gel crystallization (Fig. 1 ▸). We started with the original nano-crystallization vapour-diffusion setup (Nievergelt *et al.*, 2018 ▸), during which the cationic compound to be crystallized is screened against 96 different anion solutions simultaneously in a 96-well plate. These sitting-drop crystallizations, each requiring a volume of only 100–500 nl or even less of the porphyrin solution, are set up by a pipetting robot. With these small volumes, only 12–55 µl in total of the saturated porphyrin solution is required for setting up 96 different crystallization trials. As a result of these screenings, [5-(4-carb­oxy­phenyl)-10,15,20-tris(1-methylpyridin-1-ium-4-yl)porphyrin]^3+^ ([TriMCOOP]^3+^) yielded single crystals with benzene­sulfonate as the counter-anion, for which diffraction data could be measured directly on our home X-ray microsource diffractometer to the desired resolution of 0.78 Å. The [TriMCOOP]^3+^ cation crystallized together with three benzene­sulfonate anions (also abbreviated as besylate). This implies that the carb­oxy­lic acid group is protonated (uncharged) and indeed this assumption could be confirmed crystallographically (Supplementary Figure S7).

**Figure 3:**
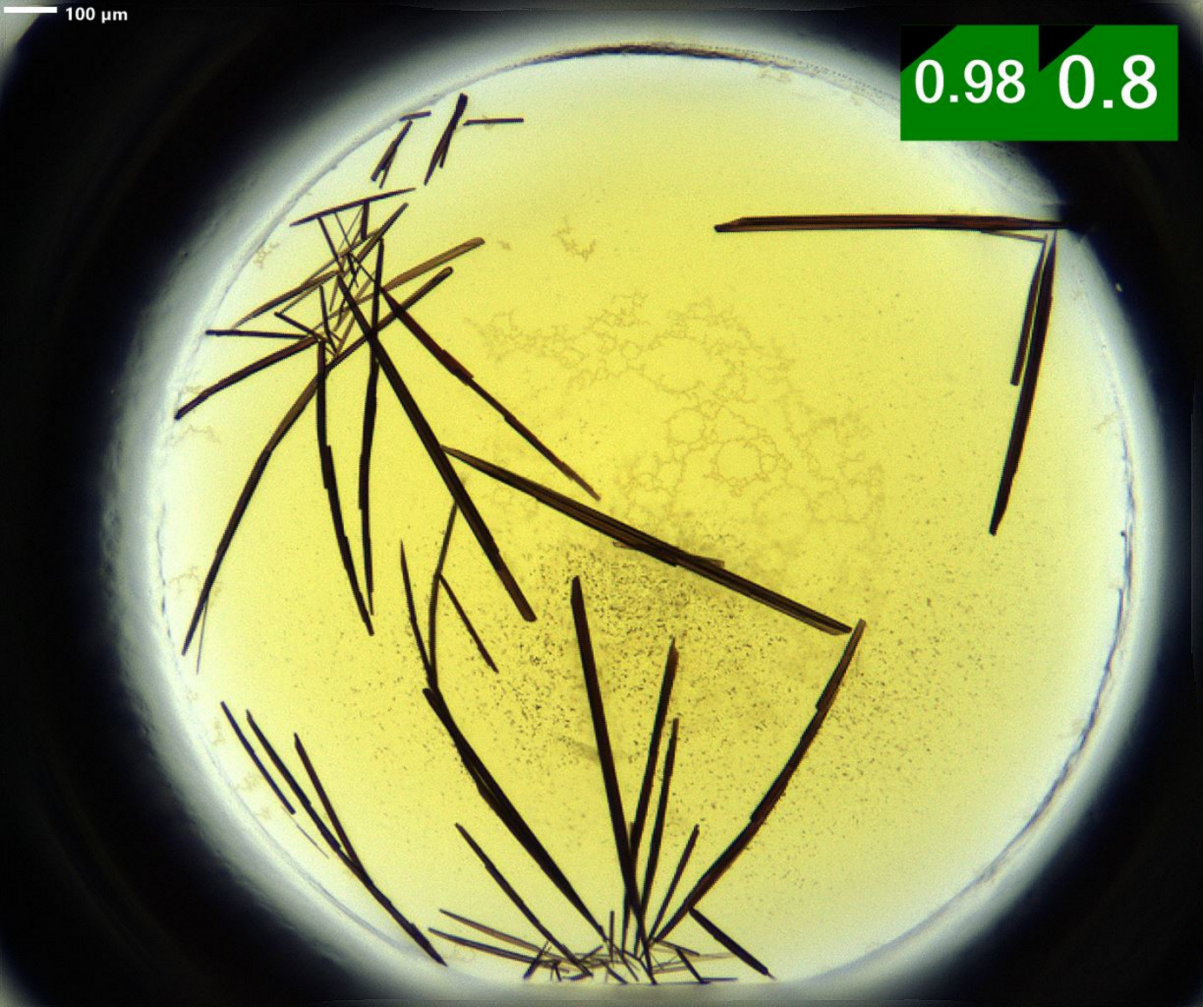
Image of one of the 96 high-throughput crystallization experiments in the crystal farm after 10 days. Shown is the crystallization using [TriMCNP](NO_3_)_3_ and 0.15 *M* sodium *p*-toluene­sulfonate. In the top-right corner, the scoring values of the MARCO and Sherlock auto-scoring models, respectively, of the *ROCK MAKER* software (Formulatrix, Dubai) are shown. The scale bar (top left) has a length of 100 µm.

The tetracationic [5,10,15,20-tetrakis(1-methylpyridin-1-ium-4-yl)porphyrin]^4+^ ([TMPyP]^4+^) was found to form crystalline material with eight different anions (see Table S1). However, the size and quality of these crystals were found to be insufficient for X-ray single-crystal analysis. On the other hand, one suitable single crystal was found for [TriMCNP]^3+^ (Fig. 3 ▸), which showed some diffraction (see below). Previously, [TMPyP](*p*-tosyl­ate)_4_ (Ford *et al.*, 1987 ▸) and several [TMPyP]^4+^ salts of a calixarene (Di Costanzo *et al.*, 2001 ▸; Gulino *et al.*, 2006 ▸) have been crystallized with the help of the vapour-diffusion technique, which is also used for protein crystallization.

Therefore, in order to improve the size and quality of the single crystals that were obtained, the ratios of anions to porphyrin cations were optimized, and after that the impact of neutral organic additives was investigated, both in EasyXtal 15-well plates (Fig. 1 ▸). Individual wells of these plates have a reservoir with a capacity of 500 µl. The wells are closed airtight to prevent evaporation of the solvents, which results in the crystallization drop to be finally located on the lower side of the closing cap as a hanging drop. With this setup, screening with different concentrations of anion solutions is easy, and efficient timewise. Furthermore, it is possible to include different organic solvents as additives, which can help to prevent unwanted crystallizations in the experiment that can be detrimental to the crystal size and quality obtained.

**Figure 4:**
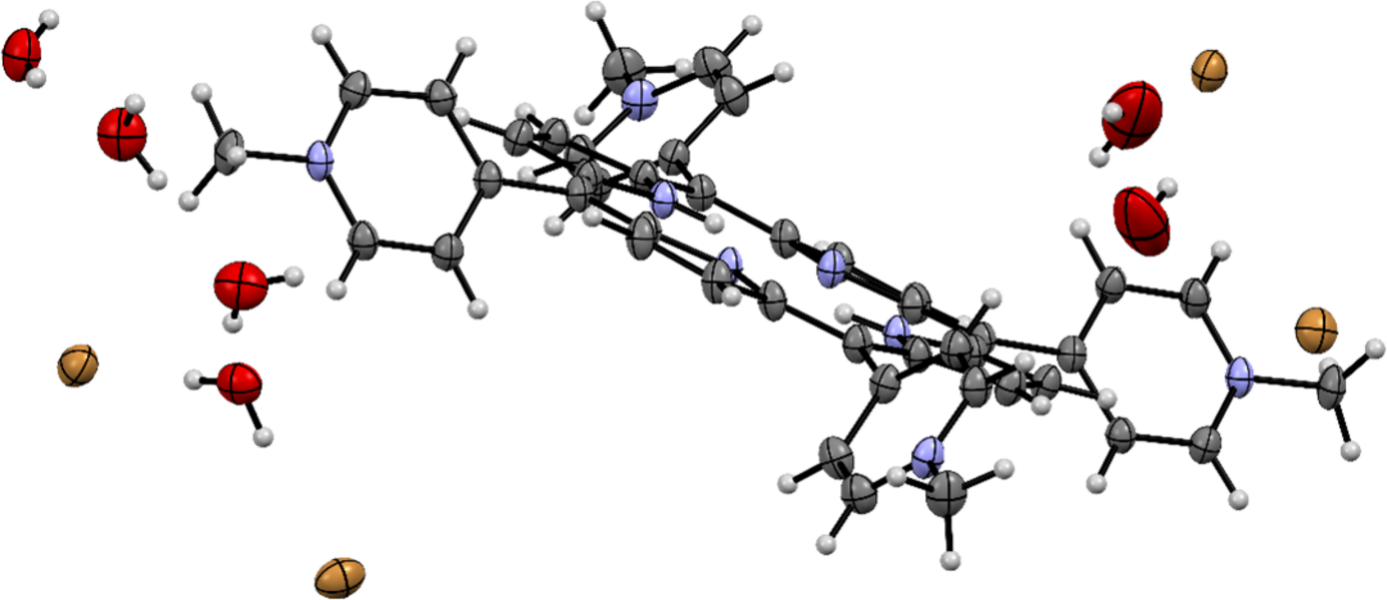
Displacement-ellipsoid representation of the bromide salt of [TMPyP]^4+^. Ellipsoids are drawn at 50% probability. All atoms in the asymmetric unit apart from two of the disordered hydrogen atoms inside the porphyrin ring are shown.

The eight anions found to crystallize with [TMPyP]^4+^, but resulting in too-small crystals, were tested at lower concentrations compared with those employed in the original sitting-drop experiments by using the hanging-drop method (see Table S1). The anion salt concentrations were lowered fivefold in steps of approximately 10% versus the original conditions, as inspired by the literature (McPherson & Cudney, 2014 ▸; Dinç *et al.*, 2016 ▸). In this way, it was possible to crystallize [TMPyP]^4+^ as the naphthalene-2,6-disulfonate salt (see Figure S1) using a concentration of 0.076 *M* of naphthalene-2,6-disulfonate instead of 0.085 *M* in the original setup. The other seven anions were then used for crystallization trials via the hanging-drop method, with the incorporation of five different organic solvents as additives (see Table 1 ▸). The choice of these five additives was inspired by neutral organic solvents in the additive screen of Hampton Research (CA, USA) and additionally by previous cases that had been reported as successful. The experiments resulted in three more types of well-diffracting single crystals whose structures could be determined in publication quality. These were [TMPyP]^4+^ with either bromide having ethyl acetate as an additive, nitrate with methanol or naphthalene-1-sulfonate with dioxane (Figs. 4 ▸ and S2–S4). In the case of the [TMPyP]^4+^ tetrabromide crystal structure, it was shown with the help of the software *Platon* (Spek, 2023 ▸) that the refined model for the structure contains no voids. In other words, apart from the six clearly identified water molecules, the organic additive (ethyl acetate) could not be identified in the crystal structure. The [TMPyP]^4+^ cation has previously been crystallized as the iodide salt (Lourenço *et al.*, 2011 ▸). However, the bromide salt described here, which is a hexahydrate crystallizing in the space group *P*1, is not isostructural with its iodide analogue, which was a tetrahydrate that crystallized in the monoclinic space group *P*2_1_/*n*.

No diffraction-quality crystals of [TMPyP]^4+^ with either di­phenyl acetate, (+)-*O*,*O*′-di­benzoyl-d-tartrate or di­hydrogen phosphate as the anion were obtained, even after optimization of the anion-to-cation ratio and tests with organic additives. Finally, improvement of the crystallization of the *p*-toluene­sulfonate salt of [TMPyP]^4+^ was attempted by employing gel crystallization (see Section 3.5). The use of agarose as a gelling agent resulted in the formation of a non-crystalline compound, which was not further analysed. Additionally, gel crystallization using TMOS did not result in the formation of any crystals or other solid material (Table 2 ▸).

Comparing the crystallographic data for the crystals containing 5,10,15,20-tetrakis(1-methylpyridin-1-ium-4-yl)porphyrin ([TMPyP]^4+^) and different counter anions (Table S4), one can see that all crystallized in the triclinic space group *P*1. Additionally, all four crystal structures containing [TMPyP]^4+^ have an asymmetric unit containing one formula unit, although in the case of the nitrate and naphthalene-1-sulfonate salts there are two symmetry-independent halves of porphyrin cations, which are completed by the action of the inversion centre.

**Figure 5:**
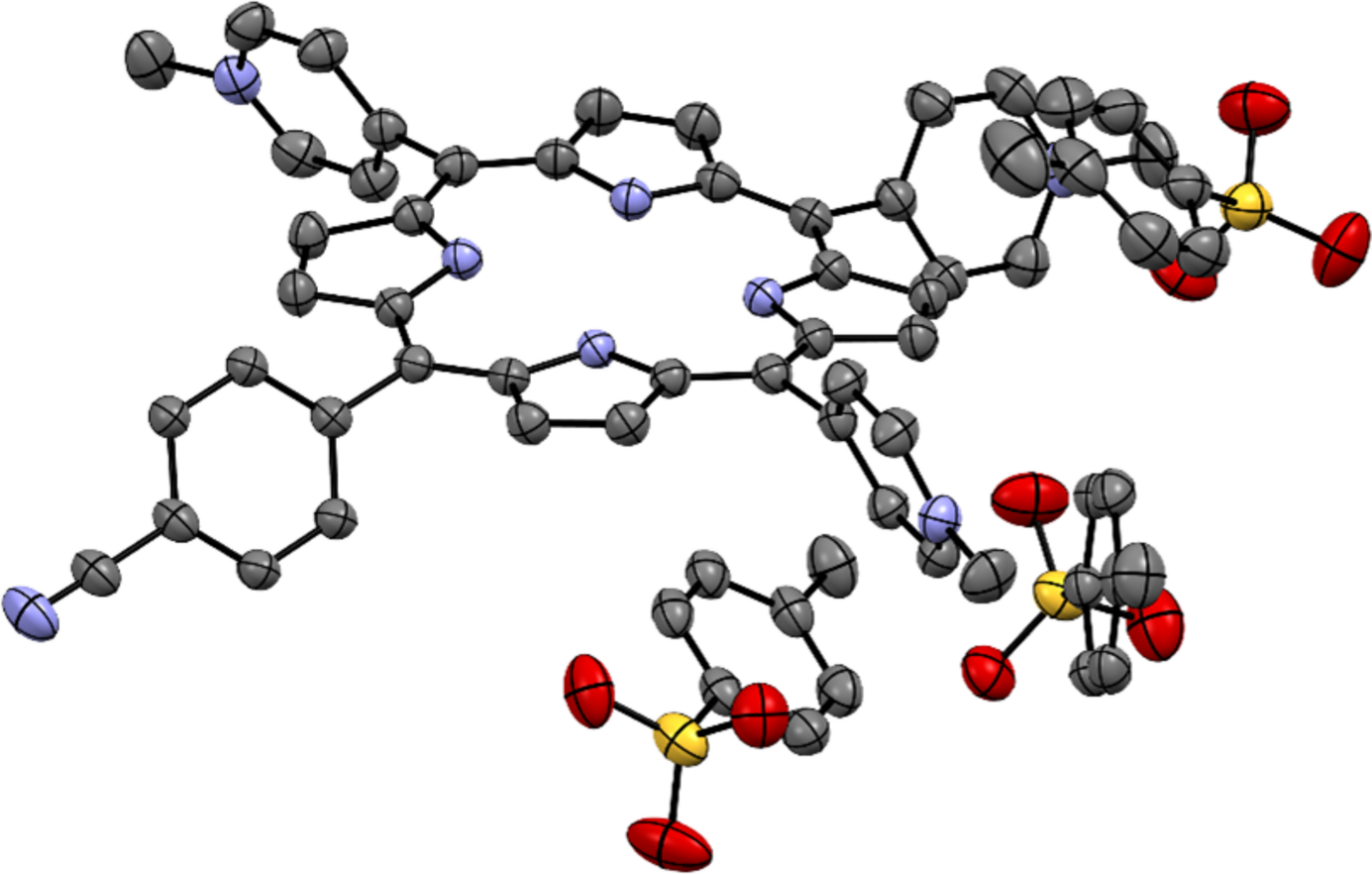
Displacement-ellipsoid representation of the hydrated *p*-toluene­sulfonate salt of [TriMCNP]^3+^ grown by gel crystallization. Ellipsoids are drawn at 50% probability. Only one porphyrin molecule out of two in the asymmetric unit and the corresponding three tosyl­ate anions are shown. All hydrogen atoms, the minor part of a disordered phenyl­ene ring and water molecules are omitted for clarity.

As already mentioned, crystallization of the tricationic 5-(4-cyanophenyl)-10,15,20-tris­(1-methylpyridin-1-ium-4-yl)­porphyrin [TriMCNP]^3+^ was also tested with the nano-crystallization screen (Nievergelt *et al.*, 2018 ▸). Elongated needle-shaped crystals of [TriMCNP](*p*-tosyl­ate)_3_·(H_2_O)_8.75_ (Fig. 3 ▸) were obtained with 0.15 *M* sodium *p*-toluene­sulfonate. These crystals, however, were quite small and only diffracted to a resolution of 1.05 Å (see Tables 2 ▸ and S5). Therefore, gel crystallization was employed in an attempt to improve the quality of diffraction. The crystallization experiments were conducted either using 0.5%(*m*/*v*) agarose (Gavira & García-Ruiz, 2002 ▸) or 9%(*v*/*v*) TMOS (Pasero *et al.*, 2025 ▸) as a gelling agent. The limited material available did not allow the gellant concentration or the curing temperature to be varied systematically. As in the case of [TMPyP]^4+^, the gel crystallization using TMOS did not result in the formation of any [TriMCNP]^3+^-containing crystals or other solid material. In contrast, the agarose gel crystallization experiment yielded after several weeks good, large single crystals of the [TriMCNP]^3+^*p*-toluene­sulfonate salt (Table 2 ▸, Fig. 5 ▸) with the same space group and very similar unit-cell dimensions as the crystals obtained by vapour diffusion (Table 3 ▸). These crystals were almost 13 times larger than those from the sitting-drop vapour diffusion crystallization and diffracted to a much better resolution (0.78 versus 1.05 Å). Consequently, a higher-quality crystal structure with lower *R*_int_ and *R*1 values was obtained by employing the agarose gel crystallization. The higher resolution also led to the clear elucidation of the positions of more disordered water molecules, although not all of them could be modelled in a satisfactory way [see Nittinger *et al.* (2015 ▸) for a related discussion about the water molecules in ultra-high-resolution protein structures]. As a consequence, the *SQUEEZE* procedure within *Platon* had to be used (Spek, 2015 ▸), thereby lowering the number of clearly identifiable water molecules despite the higher resolution. Both structures contain two formula units in the asymmetric unit. To the best of our knowledge, this is the first crystal structure of any compound containing the 5-phenyl-10,15,20-tris(pyridyl)­porphyrin core unit. It is interesting to note that porphyrins with 1-methylpyridin-1-ium-4-yl substituents at the *meso* positions apparently have a strong propensity to form crystalline materials with aryl sulfonates, as four out of the six structures reported here contain aryl sulfonates. Using the nano-crystallization technique results in an essentially unbiased testing of 77 diverse anions, out of which only six are aryl sulfonates (so only 7.8% of all tested anions). The crystal structures containing the [TMPyP]^4+^ cation with the four different anions correspond to a success rate of 5.2% with respect to the 77 anions that were used, while for both [TriMCNP]^3+^ and [TriMCOOP]^3+^ only one anion yielded usable crystals, corresponding to a success rate of 1.3%.

**Figure 6:**
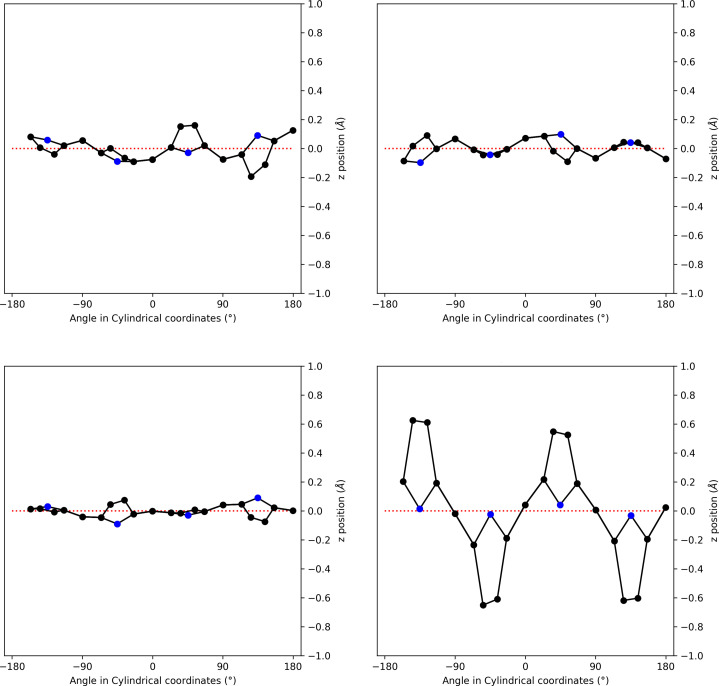
Out-of-plane distortion of the four studied [TMPyP]^4+^ salts calculated using the NSD server (Kingsbury & Senge, 2021 ▸). From top left to bottom right are shown: the bromide, nitrate (first molecule), naphthalene-1-sulfonate (first molecule) and the naphthalene-2,6-disulfonate salts. Complete data can be found in the supporting information.

**Figure 7:**
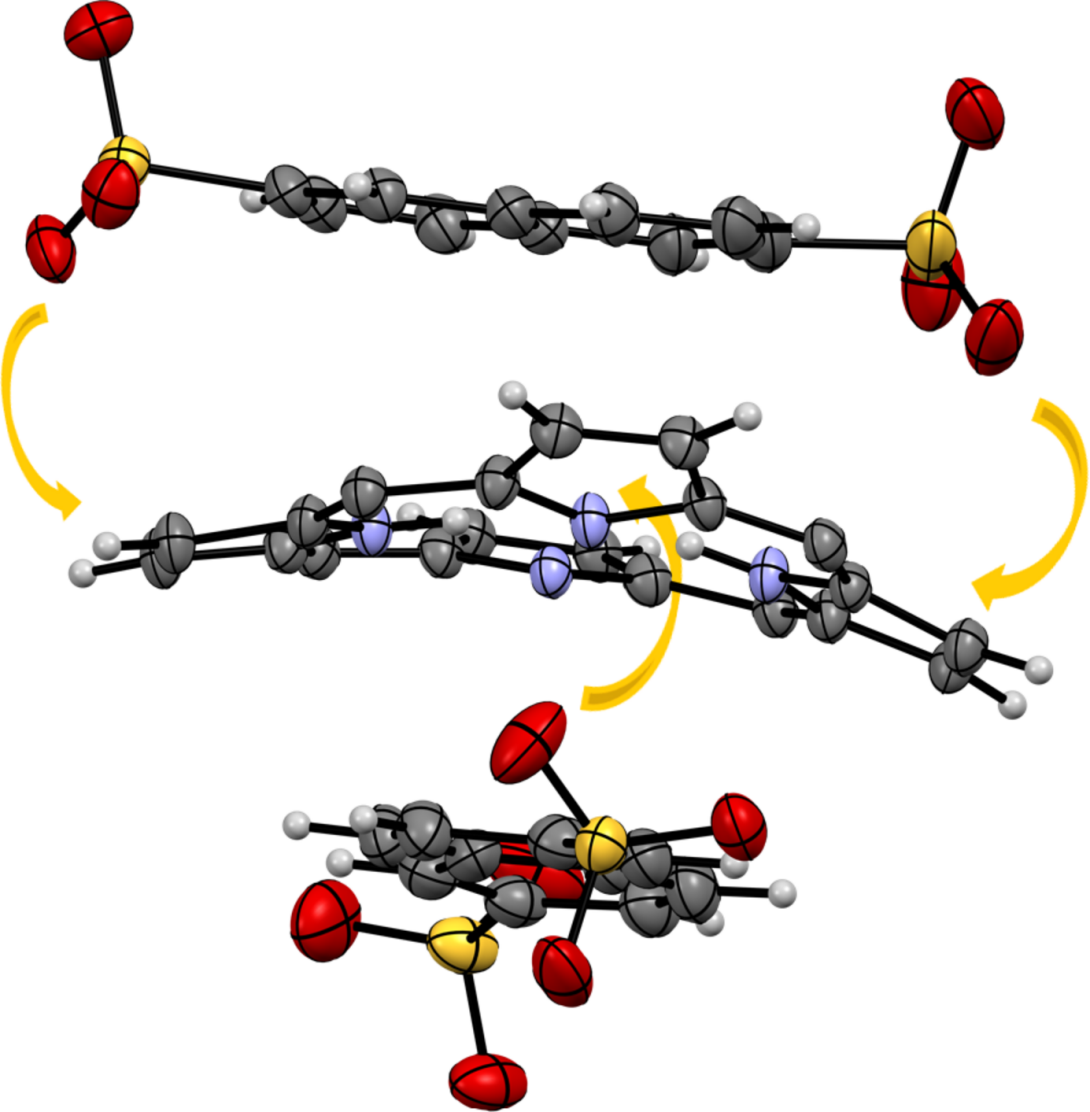
Explanation of the extreme out-of-plane distortion of the cation in the [TMPyP]^4+^ naphthalene-2,6-disulfonate salt. The *meso* substituents of the porphyrin, minor disordered parts and all solvent molecules have been omitted for clarity.

We used the availability of four crystal structures of [TMPyP]^4+^ with identical cationic porphyrin units and different anions to evaluate the influence of the anions and solvent molecules upon the in-plane and out-of-plane distortions of the porphyrins. For these calculations, we employed the normal-coordinate structural decomposition (NSD) method, which is readily available at the online server provided by the Senge group at https://www.sengegroup.eu/nsd (Kingsbury & Senge, 2021 ▸). The results of these calculations can be found in the supporting information. The in-plane distortions are small for all structures; the out-of-plane distortions are rather moderate for all structures, except for the striking case of the naphthalene-2,6-disulfonate salt (Fig. 6 ▸). As discussed in detail by Kingsbury and Senge, the causes of the porphyrin-ring distortion are mainly of an intrinsic nature, such as protonation or alkyl­ation of the inner nitro­gen atoms of the porphyrin ring, metal coordination or the degree of non-hydrogen-atom substitution at the peripheral positions. For the four structures under consideration here, all porphyrin rings are chemically identical, therefore only extrinsic packing effects can account for the remarkable out-of-plane distortion observed in case of the naphthalene-2,6-disulfonate salt. We explain this distortion by the steric impact of the dumbbell shape of the naphthalene-2,6-disulfonate anions, which lie above and, turned by 90°, below the mean porphyrin plane (Fig. 7 ▸). The bulky sulfonate groups at both ends of the anion push the parallel-aligned and *trans*-disposed pyrrole rings out of the plane.

## 3. Materials and methods

### 3.1. Materials

[TMPyP]Cl_4_ was bought from PorphyChem (Longvic, France). [TriMCNP](NO_3_)_3_ was synthesized as described in Antoni *et al.* (2015 ▸). [TriMCOOP]Cl_3_ was a generous donation from PorphyChem. The 15-well plates that were used were from Qiagen (Zug, Switzerland, article number 55408554). The 8 ml glass screw-cap vials were bought from Huberlab (Aesch, Switzerland, product number 9.7611.50). The anion screen was purchased from Molecular Dimensions (Calibre Scientific, Los Angeles, CA, USA).

### 3.2. Crystallization using semi-automatic crystallization

The crystallization screening experiments were performed with the help of the Gryphon LCP nano-drop handler from Art Robbins Instruments in ARI Intelli-Plates 96-3 LVR. 100–500 nl of a stock solution of the to-be-crystallized cation were mixed with the same volume of the stock solutions of the salt of the counterion and equilibrated against 75 µl of the stock solution of the same counterion. Each cation was tested for crystallization in 96 wells, each well containing a different condition, and with 77 different anions in total. Owing to the high viscosity of some solutions, all pipetting was done with slow speed. Plates were incubated for 5–16 days at 20°C. The Rock Imager 1000 took a picture of each well with normal light (immediately after setting up the plate and then after 2, 5, 10 and 16 days) and cross-polarized light (Nievergelt *et al.*, 2018 ▸). The porphyrin stock solutions used were saturated aqueous solutions.

### 3.3. Optimization of the crystallization of porphyrin cations using hanging-drop crystallization and varying the ratio of cation to anion

Using a 15-well plate, sodium salt solutions (500 µl) with the concentrations given in Table S1 were added into the reservoirs. On the inner side of the seal, a drop of a saturated aqueous [TMPyP]Cl_4_ porphyrin solution (1.0 µl) was added using an Eppendorf pipette. A drop of the anion solution (1.0 µl) from the reservoir was added into the drop of the porphyrin solution. The seal was then carefully put onto the well, which was closed tightly to prevent evaporation of the liquids. The plate was then left undisturbed for crystal growth. For each anion, five different concentrations of anions with lower concentrations than those used in the original crystallizations described in Section 3.2 were employed in order to slow down the crystal growth (Table S1).

### 3.4. Optimization of the crystallization of porphyrin cations with organic neutral additives using hanging-drop crystallization

Into the reservoirs of a 15-well plate, a sodium salt solution (450 µl; in the case of ethyl acetate 475 µl) with the concentration listed in Table S2 was added. To this sodium salt solution, an aqueous solution of an organic additive (50 µl, or pure ethyl acetate 25 µl) was added, as listed in Table S3. The well solution in the reservoir was mixed. On the inner side of the seal a drop of a saturated aqueous [TMPyP]Cl_4_ porphyrin solution (1.0 µl) was added using an Eppendorf pipette. A drop of the anion solution (1.0 µl) from the reservoir with the organic additive was added to the drop of the porphyrin solution. The seal was then carefully put onto the well, which was closed tightly to prevent evaporation of the liquids. The plate was then left undisturbed for crystal growth.

### 3.5. Preparation of porphyrin-cation-containing crystals using gel crystallization

**Figure 8:**
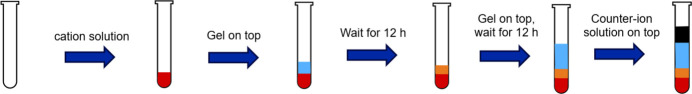
Setup of the gel crystallization by counter-diffusion.

In 1.5 ml vials for gas chromatography (GC), aqueous solutions of either [TriMCNP](NO_3_)_3_ (76.1 mg in 1.00 ml) or [TMPyP]Cl_4_ (100 mg in 1.00 ml) were prepared. In a 50 ml glass beaker, Millipore water (20.0 ml) was added. Agarose (100 mg) was added to the water. The mixture was then stirred and heated to 85°C using a heating plate. In a 50 ml plastic centrifuge tube, water (2.00 ml) was added together with tetra­methyl orthosilicate (TMOS, 0.20 ml). This solution was stirred for 15 minutes. Either a [TriMCNP](NO_3_)_3_ (0.50 ml) or a [TMPyP]Cl_4_ (0.50 ml) solution was carefully injected into the bottom of an 8 ml screw-cap test tube with a syringe. On top of the solution, either not-yet solidified TMOS (0.50 ml) or agarose gel (0.50 ml) solutions were layered using a syringe. The test tubes were then closed and the gel left to solidify overnight. After the gel had solidified, another layer of the corresponding freshly prepared gel solution (1.50 ml) was carefully layered on top of the first layer, to prevent mixing of the two layers. After the second gel layer had solidified, again overnight, a sodium *p*-toluene­sulfonate (0.15 *M*, 0.50 ml) solution was carefully layered on top of the first two layers using a syringe. The schematic setup is shown in Fig. 8 ▸.

### 3.6. Calculation of the in-plane and out-of-plane distortions of the porphyrins

The atomic coordinates of each crystal structure in CIF format were read into *Mercury* (Macrae *et al.*, 2020 ▸), all anions and water molecules were removed and the remaining atom list was saved as in the Protein Data Bank (.pdb) file format. In the case of the naphthalene-2,6-disulfonate salt, the *meso* substituents of the porphyrin had to be removed for the calculations. The .pdb files were read into the NSD server at https://www.sengegroup.eu/nsd (Kingsbury & Senge, 2021 ▸).

## 4. Conclusions

In this paper, we have described a systematic approach for obtaining high-quality crystals of salts of the polycationic, water soluble porphyrins [TMPyP]^4+^, [TriMCOOP]^3+^ and [TriMCNP]^3+^. Starting from the nano-crystallization anion screen, which yielded high-quality crystals of one compound for structure determination and other initial crystalline hits, the latter could be improved by manually optimizing the stoichiometric ratio between the anion and cation. Taking the best conditions from this optimization, three more publication-quality single-crystal structures of porphyrin salts were obtained using neutral organic additives. These additives helped to improve the crystallization without being incorporated into the crystal structures. As an additional method for challenging cases, gel crystallization was used successfully for the optimization of one system, leading to the determination of a higher-quality crystal structure with lower *R* values together with an improvement of the diffraction resolution from 1.05 to 0.78 Å. To the best of our knowledge, the present publication is the first report of the crystallization of a porphyrin with the help of a gel, as well as the first crystal structure of an *A*_3_*B* porphyrin with the 5-phenyl-10,15,20-tris(pyridyl)­porphyrin core unit.

**Figure 9:**
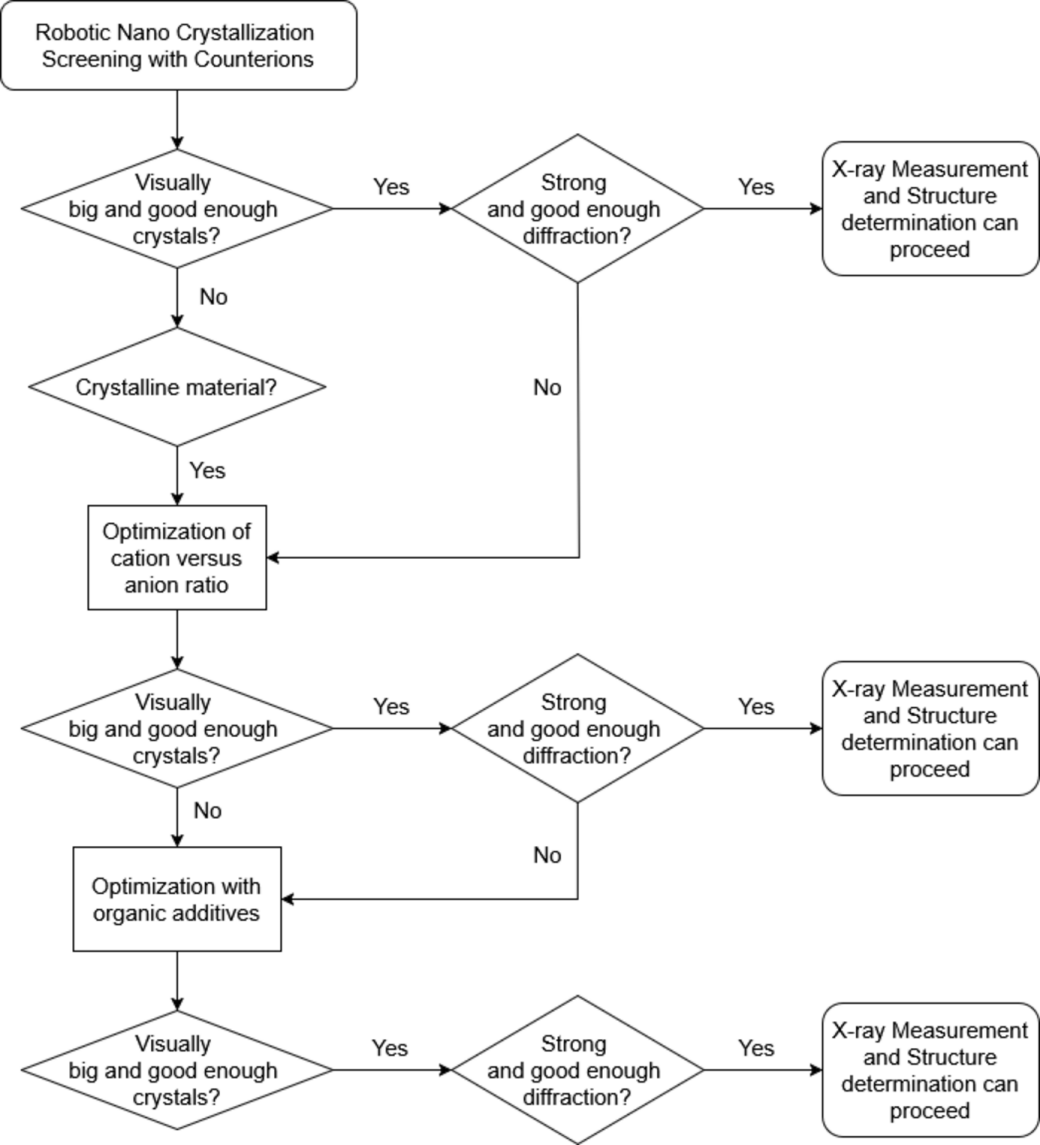
Flow diagram for the nano-crystallization screen. The flow diagram was drawn with the help of diagrams.net (https://www.diagrams.net) by JGraph, 2025, version 28.2.7.

The proposed decision tree for the nano-crystallization screen is summarized in Fig. 9 ▸.

Finally, normal-coordinate structural decomposition (NSD) analysis revealed a rare example of an extrinsic distortion of a porphyrin ring induced by the dumbbell-shaped naphthalene-2,6-disulfonate anions above and, perpendicular to the first anion, below the porphyrin ring.

## 5. Related literature

The following references are cited in the supporting information: Dolomanov *et al.* (2009 ▸), Rigaku Oxford Diffraction (2024 ▸), Sheldrick (2015*a* ▸, 2015*b* ▸) and Thorn *et al.* (2012 ▸).

## Supplementary Material

Crystal structure: contains datablock(s) (TMPyP)(nitrate)4(H2O)2.5, (TMPyP)(2,6-naphthalenedisulfonate]2(H2O)7.5, (TMPyP)(1-naphthalenesulfonate)4, (TriMCNP)(p-tosylate)3(H2O)8.75, (TriMCNP)(p-tosylate)3(H2O)7, (TriMCOOP)(besylate)3(H2O)4, (TMPyP)Br4(H2O)6. DOI: 10.1107/S2052252525009716/yc5052sup1.cif

Supporting information. DOI: 10.1107/S2052252525009716/yc5052sup2.pdf

CCDC references: 2465390, 2465391, 2465392, 2465393, 2465394, 2465395, 2465396

## Figures and Tables

**Table 1:** Optimization of the aqueous crystallization of [TMPyP]^4+^ using organic additives

Organic additive	Final concentration in volume % of organic additive in reservoir solution	Previously reported by
1,4-Dioxane	5	Cudney *et al.*, 1994 ▸; Rohner *et al.*, 2016 ▸
Methanol	5	Luberacki *et al.*, 2008 ▸
Aceto­nitrile	5	Gosavi *et al.*, 2009 ▸
Ethyl acetate	5	Spingler *et al.*, 2001 ▸; Rohner *et al.*, 2016 ▸
Acetone	4	Nievergelt *et al.*, 2018 ▸

**Table 2:** Qualitative results of the gel crystallization of [TMPyP]^4+^ and [TriMCNP]^3+^ as their *p*-toluene­sulfonate salts

Cation and anion	0.5%(*m*/*v*) Agarose	9%(*v*/*v*) TMOS
[TMPyP]^4+^ and *p*-toluene­sulfonate	Non-crystalline compound	No solid material at all
[TriMCNP]^3+^ and *p*-toluene­sulfonate	Good quality, large crystals	No solid material at all

**Table 3:** Comparison of crystal data for [TriMCNP](*p*-toluene­sulfonate)_3_·*n*H_2_O obtained by vapour diffusion and gel crystallization

	[TriMCNP](*p*-tosyl­ate)_3_·(H_2_O)_8.75_ (vapour crystallization)	[TriMCNP](*p*-tosyl­ate)_3_·(H_2_O)_7_ (gel crystallization)
Space group	*P*2_1_/*c*	*P*2_1_/*c*
*a* (Å)	17.50074 (18)	17.54006 (8)
*b* (Å)	25.9751 (3)	25.99854 (12)
*c* (Å)	30.1002 (4)	30.05455 (13)
β (°)	100.4228 (12)	100.5017 (4)
Volume (Å^3^)	13457.2 (3)	13475.78 (10)
Crystal size (mm)	0.44 × 0.034 × 0.03	0.267 × 0.153 × 0.142
θ range (°)	2.263 to 47.692	2.263 to 79.242
Reflections collected	99768	275353
Independent reflections	12390 (*R*_int_ = 0.0470)	28905 (*R*_int_ = 0.0389)
Reflections observed	10103	25118
Data/restraints/parameters	12390/13/1812	28905/215/1758
Final *R* factors [*I*> 2σ(*I*)]	*R*1 = 0.0777, *wR*2 = 0.2123	*R*1 = 0.0715, *wR*2 = 0.2108
*R* factors (all data)	*R*1 = 0.0929, *wR*2 = 0.2275	*R*1 = 0.0780, *wR*2 = 0.2183

## Data Availability

CCDC entries 2465390–2465396 contain the supplementary crystallographic data for this paper. These data are provided free of charge by The Cambridge Crystallographic Data Centre via https://www.ccdc.cam.ac.uk/structures. Raw data are available upon request from the authors.
