# Supplementary material for: Obtaining single crystals containing cationic porphyrins from aqueous solutions: a systematic approach including nano-crystallization, organic modifiers and gel crystallization
Source: IUCrJ. 2026 Jan 1;13(Pt 1):44–52. doi: 10.1107/S2052252525009716 (PMC12809503; doi:10.1107/S2052252525009716)
Supplement: Supplementary file 2 [file m-13-00044-sup2.pdf]

IUCrJ

Volume 13 (2026)

Supporting information for article:

Obtaining single crystals containing cationic porphyrins from aqueous solutions: a systematic approach including nano-crystallization, organic modifiers and gel crystallization

Rayk A. Schmitz, Florian C. Brunner, Leonard P. Zelder and Bernhard Spingler

Supplementary Table S1 Sodium salt solution concentrations in the corresponding wells for the optimization of the crystallization of salts of **[TMPyP]$^{4+}$**. The successful crystallization setup to give single crystals suitable for single crystal structure analysis is highlighted in bold.

**compound**	**conc. well 1 [M]**	**conc. well 2 [M]**	**conc. well 3 [M]**	**conc. well 4 [M]**	**conc. well 5 [M]**
sodium bromide	1.80	1.60	1.40	1.20	1.00
sodium 1-naphthalenesulfonate	0.30	0.28	0.26	0.24	0.22
disodium 2,6-naphthalenesulfonate	0.080	0.078	**0.076**	0.074	0.072
sodium nitrate	2.10	1.90	1.70	1.50	1.30
sodium diphenyl acetate	0.30	0.28	0.26	0.24	0.22
disodium (+)-*O,O'*-dibenzoyl-D-tartrate	0.24	0.22	0.20	0.18	0.16
sodium dihydrogen phosphate	1.80	1.60	1.40	1.20	1.00
sodium *p*-toluenesulfonate	0.15	0.12	0.09	-	-

Supplementary Table S2 Sodium salt and concentration of aqueous solutions used for Hanging-Drop Crystallization of [**TMPyP**]Cl_4 with organic additives.

**compound**	**conc. [M]**
sodium bromide	1.00
sodium 1-naphthalenesulfonate	0.22
sodium nitrate	1.30
disodium (+)-*O,O'*-dibenzoyl-D-tartrate	0.20
sodium dihydrogen phosphate	1.40

Supplementary Table S3 Organic additives and their concentrations in aqueous solutions.

well	organic additive	conc. [% v/v]
1	1,4-dioxane	50
2	methanol	50
3	acetonitrile	50
4	ethyl acetate	*
5	acetone	40

*5% v/v of pure ethyl acetate was used

Procedure for the Single Crystal X-ray Diffraction Measurements

Crystallographic data were collected on a Rigaku Oxford Diffraction XtaLAB Synergy-S dual source diffractometer fitted with a Dectris Pilatus3 R 200K HPC (Hybrid Photon Counting) detector, Cu and Mo PhotonJet microfocus X-ray sources, and an Oxford Cryosystems Cryostream 800 cooler maintaining a temperature of 160.0(1) K. Suitable crystals were covered with oil (Infineum V8512, formerly known as Paratone N), placed on a nylon loop that is mounted on a CrystalCap Magnetic™ pin (Hampton Research) and immediately transferred to the diffractometer. The program suite *CrysAlis*^{Pro} was used for data collection, numerical or multi-scan absorption corrections, as well as data reduction (Rigaku Oxford Diffraction, 2024). Each structure was solved with the dual-space algorithm using *SHELXT* (Sheldrick, 2015a) and was refined by full-matrix least-squares methods on F^2 with *SHELXL-2018* (Sheldrick, 2015b) using the *Olex2* GUI (Dolomanov *et al.*, 2009) (Tables S4 and S5). Ill-defined electron density had to be treated with the *Squeeze* procedure within *Platon* (Spek, 2015), see Tables S4 and S5. The graphical output was produced with the help of the program *Mercury* (Macrae *et al.*, 2020).

Description of Crystal Structures

[TMPyP]·[2,6-naphthalenedisulfonate]$_2$·$(H_2O)_{7.5}$

Crystalline material was obtained from the hanging-drop crystallization [from a drop containing 1.0 µl of a 0.076 M disodium 2,6-naphthalenedisulfonate aqueous solution and 1.0 µl of a saturated aqueous 5,10,15,20-tetra(*N*-methyl-4-pyridinium)porphyrin chloride solution equilibrating against a reservoir of a 0.078 M disodium 2,6-naphthalenedisulfonate aqueous solution. Tetracationic 5,10,15,20-tetra(*N*-methyl-4-pyridinium)porphyrin crystallized with two dianionic 2,6-naphtalenedisulfonate counter-ions as a polyhydrate in the triclinic space group $P\overline{1}$. The asymmetric and formula unit are built up by one tetravalent 5,10,15,20-tetra(*N*-methyl-4-pyridinium)porphyrin cation, two 2,6-naphthalenedisulfonate anions and 8.5 water molecules. The structural formula in the refinement model is $C_{44}H_{38}N_8$, $2(C_{10}H_6O_6S_2)$, $7.5(H_2O)$, HO (the location of the second hydrogen atom of one water molecule could not be found). Due to non-merohedral twinning, which was partly recognized and treated with a twin law found by *Platon* (Spek, 2023), the positions of the hydrogen atoms of the water molecules were difficult to refine and had to be partly restrained with bond length restraints (DFIX instructions). One sulfonate group is disordered in a ratio of 84:16. Within the porphyrin ring, hydrogen bonds are formed between the nitrogen atoms (N2-H2…N3: 2.885(6) Å). The 2,6-naphtalenedisulfonate molecules form hydrogen bonds from their sulfonate groups to adjacent free water molecules [O13-H13B O10: 2.767(8) Å; O14-H14A O7: 2.84(1) Å; O17-H17A O1: 2.71(1) Å; O18-H18A O3: 2.79(1) Å; O19-H19B O6: 2.962(9) Å, O16-H16A O12: 2.978(9) Å;]. The free water molecules also form hydrogen bonds with each other [O13-H13A O18: 2.77(1) Å; O14-H14B O15: 2.91(1) Å; O15-H15A O13: 2.738(8) Å; O15-H15B O16: 2.75(1) Å; O16-H16B O15: 2.81(1) Å; O18-H18B O19: 2.81(1) Å; O19-H19A O17: 2.65(2) Å; O20-H20A O21: 2.92(3) Å; O20-H20A O21: 2.67(3) Å; O20-H20B O18: 2.74(1) Å]. Views of the structure can be seen in Supplementary Figure S1.

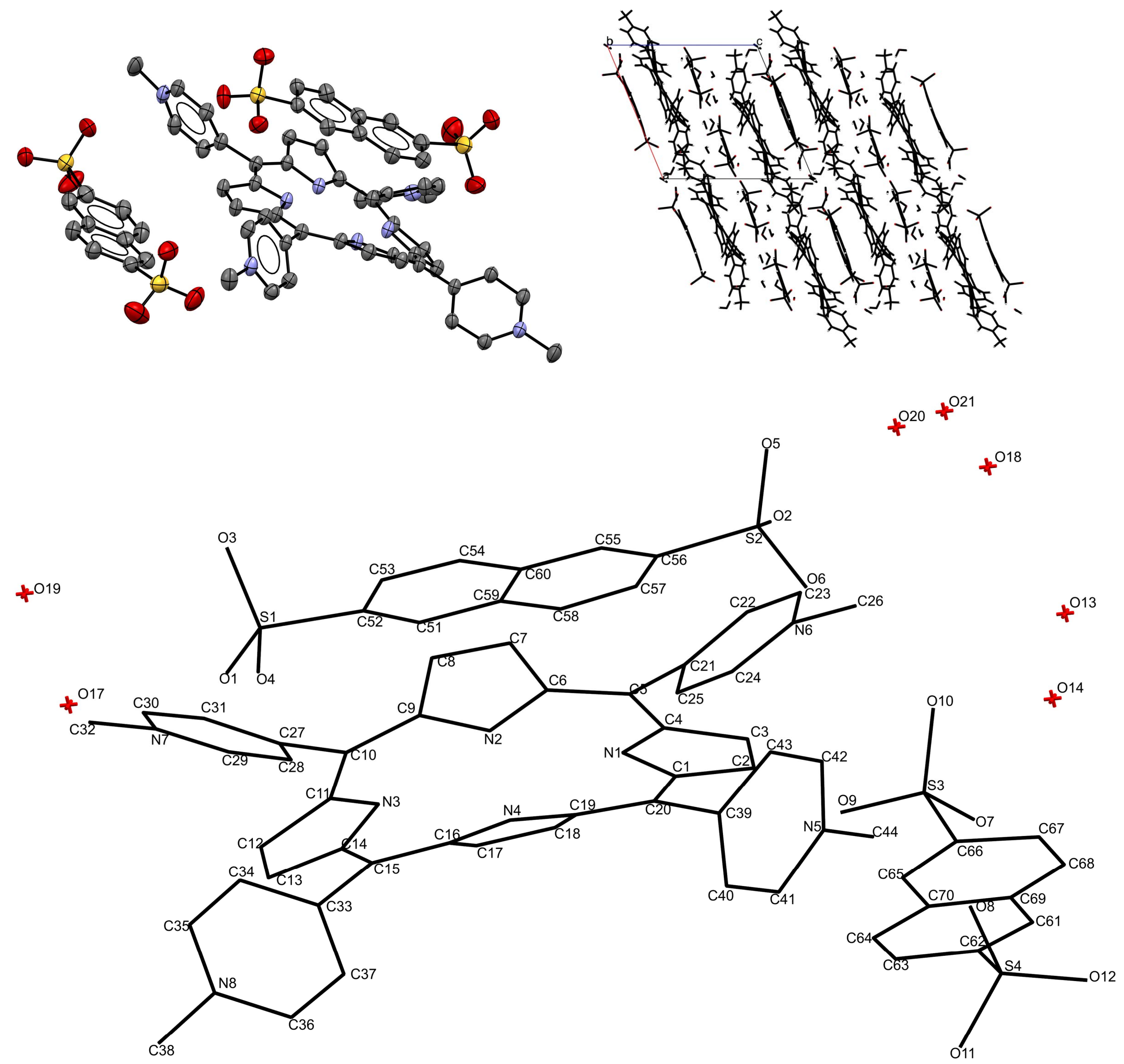

Supplementary Figure S1

Top left: Displacement ellipsoid representation of **[TMPyP]·[2,6-naphthalenedisulfonate]$_2$·(H$_2$O)$_{7.5}$**. Ellipsoids are drawn at 50% probability. All hydrogen atoms, minor disordered parts and water molecules are omitted for clarity.

Top right: Packing diagram of **[TMPyP]·[2,6-naphthalenedisulfonate]$_2$·(H$_2$O)$_{7.5}$**, viewed down the [010] direction.

Bottom: Atom-labelling scheme for **[TMPyP]·[2,6-naphthalenedisulfonate]$_2$·(H$_2$O)$_{7.5}$**. All hydrogen atoms are omitted for clarity.

[TMPyP]·[1-naphthalenesulfonate]$_4$

Crystalline material was obtained from the hanging-drop crystallization [from a drop containing 1.0 μl of a 0.22 M sodium 1-naphthalenesulfonate aqueous solution (incorporating as an additive a 10 vol. % of a 50% aq. sol. of 1,4-dioxane) and 1.0 μl of a saturated aqueous 5,10,15,20-tetra(*N*-methyl-4-pyridinium)porphyrin chloride solution equilibrating against a reservoir of a 0.22 M sodium 1-naphthalenesulfonate aqueous solution (with 10 vol. % dioxane)]. Tetracationic 5,10,15,20-tetra(*N*-methyl-4-pyridinium)porphyrin crystallized with 1-naphthalenesulfonate anions, without incorporated solvent or additive, in the triclinic space group $P\overline{1}$. The asymmetric unit consists of two symmetry-independent halves of the tetravalent porphyrin cations, which lie about crystallographic centers of inversion, and four 1-naphthalenesulfonate anions. The structural formula is $C_{84}H_{86}N_8O_{14}S_4$. Three out of the four 1-naphthalene sulfonate anions are disordered as their full bodies. Their treatment required the application of rigid group and rigid bond restraints (AFIX 66 and RIGU instructions) (Thorn *et al.*, 2012). Views of the structure can be seen in Supplementary Figure S2.

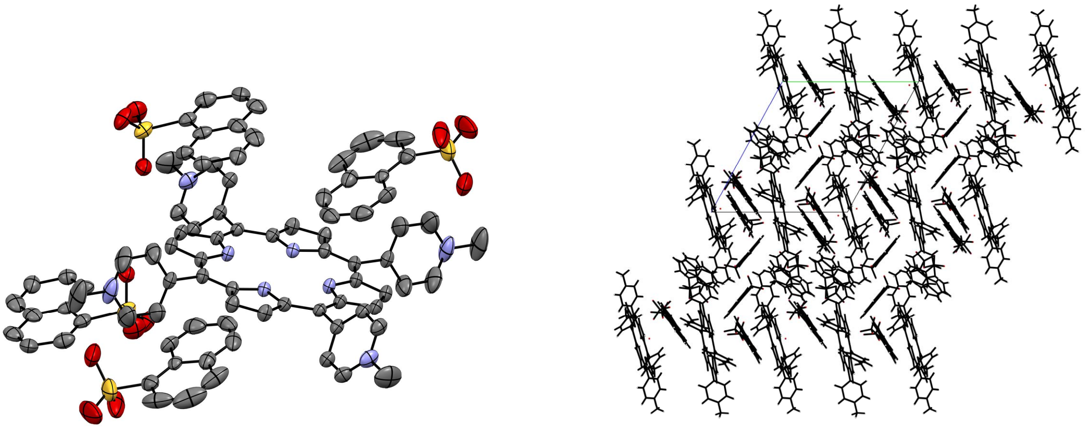

Supplementary Figure S2

Left: Displacement ellipsoid representation of **[TMPyP]·[1-naphthalenesulfonate]$_4$**. Ellipsoids are drawn at 50% probability. All hydrogen atoms, the minor parts of three disordered 1-naphthalenesulfonate anions, the second symmetry-independent porphyrin cation in the asymmetric unit and water molecules are omitted for clarity.

Right: Packing diagram of **[TMPyP]·[1-naphthalenesulfonate]$_4$**, viewed down the [100] direction.

[TMPyP]·[bromide]$_4$·(H$_2$O)$_6$

Crystalline material was obtained from the hanging-drop crystallization [from a drop containing 1.0 µl of a 1.0 M sodium bromide aqueous solution (incorporating 5 vol. % ethyl acetate as an additive) and 1.0 µl of a saturated aqueous 5,10,15,20-tetra(*N*-methyl-4-pyridinium)porphyrin chloride solution equilibrating against a reservoir of a 1.0 M sodium 1-naphthalenesulfonate aqueous solution (5 vol. % ethyl acetate)]. Tetracationic 5,10,15,20-tetra(*N*-methyl-4-pyridinium)porphyrin crystallized with four bromide counter-ions as a hexahydrate, but without incorporated additive, in the triclinic space group $P\bar{1}$. The asymmetric and formula unit comprises one tetravalent porphyrin ring cation surrounded by four bromide anions and six fully-occupied water molecules. The structural formula in the refinement model is $C_{44}H_{38}N_8$, 4Br, 5(H_2O), HO (the location of the second hydrogen atom of one water molecule could not be found). According to the software *Platon* (Spek, 2023), the unit cell does not contain any residual solvent accessible voids. The electronic structure of the porphyrin ring is disordered in a ratio 1:1, which leads to the situation that every pyrrole has a half-occupied hydrogen atom site on its nitrogen atom. Due to its position next to a centre of inversion, one of the 12 hydrogen atoms of the six water molecules could not be located. The free water molecules form hydrogen bonds among themselves [O1-H1B O2: 2.852(10) Å; O4-H4B O6: 2.833(4) Å; O6-H6A O5: 2.738(3) Å; O3-H3C O4: 2.818(4) Å]. Views of the structure can be seen in Supplementary Figure S3.

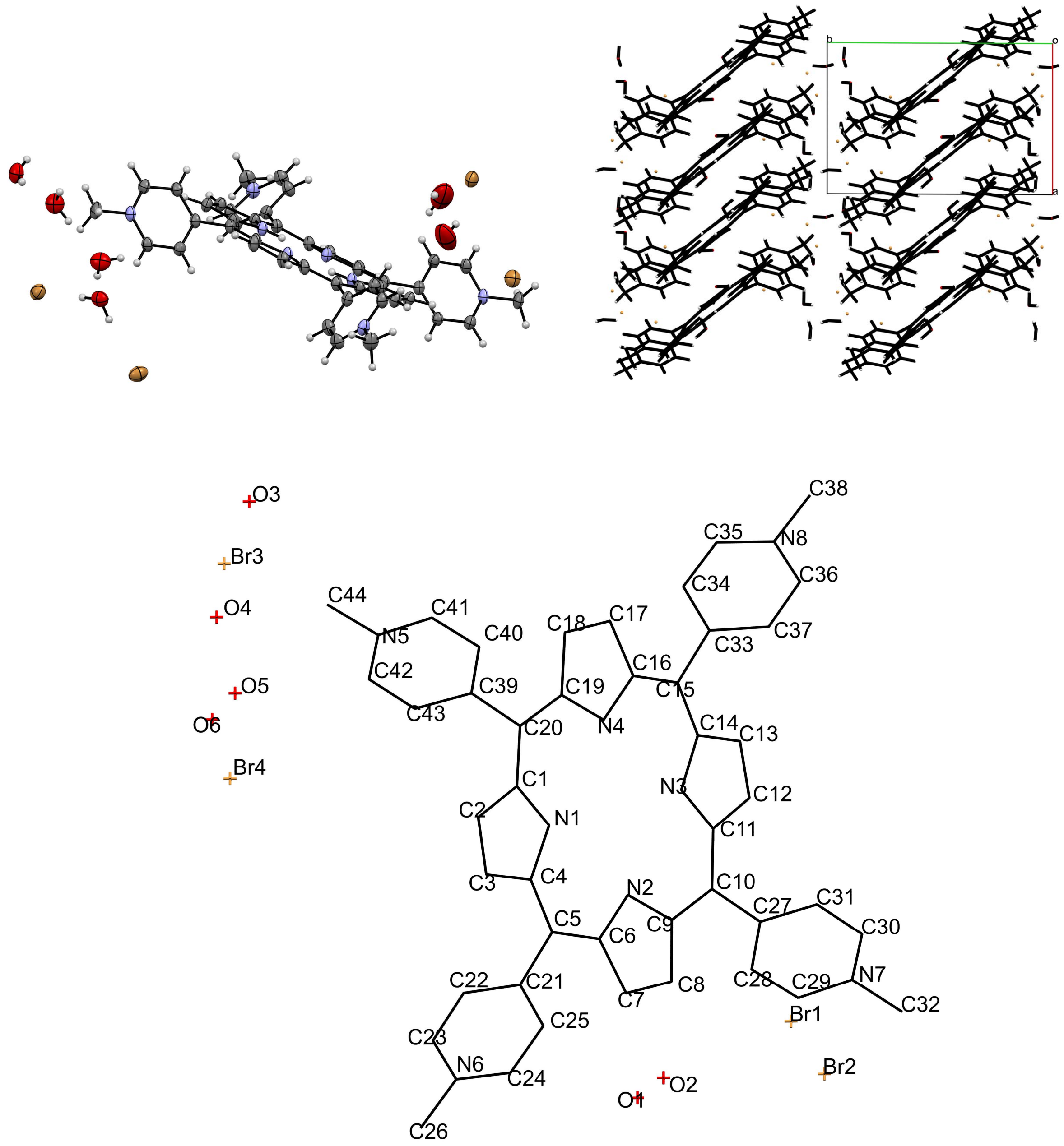

Supplementary Figure S3

Top left*:* Displacement ellipsoid representation of **[TMPyP]·[bromide]$_4$·(H$_2$O)$_6$**. Ellipsoids are drawn at 50% probability. All modelled atoms in the asymmetric unit, apart from two of the disordered pyrrole hydrogen atoms inside the porphyrin ring, are shown.

Top right: Packing diagram of **[TMPyP]·[bromide]$_4$·(H$_2$O)$_6$**, viewed down the [001] direction.

Bottom: Atom-labelling scheme for **[TMPyP]·[bromide]$_4$·(H$_2$O)$_6$**. All hydrogen atoms are omitted for clarity.

[TMPyP]·[nitrate]$_4$·(H$_2$O)$_{2.5}$

Crystalline material was obtained from the hanging-drop crystallization [from a drop containing 1.0 µl of a 1.3 M sodium nitrate aqueous solution (incorporating 10 vol. % of a 50% aq. sol. of methanol as an additive) and 1.0 µl of a saturated aqueous 5,10,15,20-tetra(*N*-methyl-4-pyridinium)porphyrin chloride solution equilibrating against a reservoir of a 1.3 M sodium nitrate aqueous solution (10 vol. % of a 50% aq. sol. of methanol)]. Tetracationic 5,10,15,20-tetra(*N*-methyl-4-pyridinium)porphyrin crystallized with nitrate anions as a polyhydrate, but without incorporated additive, in the triclinic space group $P\overline{1}$. The asymmetric unit consists of two symmetry-independent halves of the tetracationic porphyrins, which lie about crystallographic centers of inversion, four nitrate counter-ions and 2.5 molecules of water. The structural formula is $C_{44}H_{38}N_{12}O_{14.5}$. The oxygen atoms of one of the nitrate anions are disordered over two positions in a ratio 71:29, two other nitrate anions are disordered equally across two site each. Views of the structure can be seen in Supplementary Figure S4.

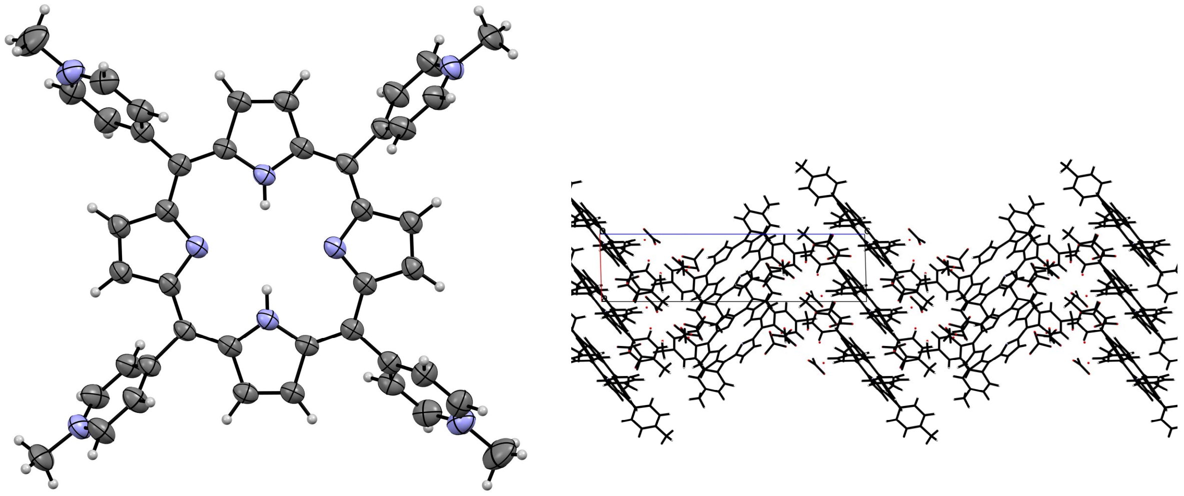

Supplementary Figure S4

Left: Displacement ellipsoid representation of one porphyrin cation in **[TMPyP]·[nitrate]$_4$·(H$_2$O)$_{2.5}$**. Ellipsoids are drawn at 50% probability. All hydrogen atoms, anions and solvent molecules are omitted for clarity.

Right: Packing diagram of **[TMPyP]·[nitrate]$_4$·(H$_2$O)$_{2.5}$**, viewed down the [010] direction.

[TriMCNP]·[*p*-tosylate]$_3$·(H$_2$O)$_{8.75}$

Crystalline material was obtained from the screening [from a drop containing 500 nl of a 0.150 M sodium *p*-toluenesulfonate aqueous solution and 500 nl of a saturated aqueous 5,10,15-tris(methylpyridinium)-20-benzonitrile-porphyrin nitrate solution equilibrating against a reservoir of a 0.150 M sodium chloride aqueous solution]. Tricationic 5,10,15-tris(methylpyridinium)-20-benzonitrile-porphyrin (TriMCNP) crystallized with *p*-toluenesulfonate anions as a polyhydrate in the monoclinic space group $P2_1/c$. The asymmetric unit comprises two trivalent 5,10,15-tris(methylpyridinium)-20-benzonitrile-porphyrin cations, six *p*-toluenesulfonate counter-ions and 17.5 free water molecules (Z' = 2). The structural formula in the refinement model is thus $C_{45}H_{35}N_8$, 3($C_7H_7O_3S$), 6(H_2O), 2.75O (the location of the hydrogen atoms of these latter water molecules could not be found). Within the porphyrin ring, hydrogen bonds are formed between the nitrogen atoms [N2-H2 N3: 2.889(7) Å; N3-H3 N2: 2.889(7) Å; N10-H10 N11: 2.887(7) Å; N10-H10 N12: 2.984(7) Å; N13-H13 N12: 2.884(7) Å]. Additionally, the cyanide group of one porphyrin ring accepts a hydrogen bond from one free water molecule (O19-H19B N8: 2.885(9) Å). All sulfonate groups acts as acceptors for two hydrogen bonds from free water molecules, except one which accepts three [O20-H20A O5: 2.824(7) Å; O20-H20B O10: 2.930(7) Å; O21-H21E O3: 2.791(8) Å; O22-H22A O16: 2.834(8) Å; O23-H23A O15: 2.755(10) Å; O23-H23B O17: 2.807(7) Å; O24-H24A O8: 2.872(7) Å; O26-H26A O12: 2.753(8) Å; O31-H31B O7: 2.926(11) Å]. The free water molecules also form hydrogen bonds among themselves [O19-H19A O21: 2.751(7) Å; O21-H21D O26: 2.789(9) Å; O22-H22B O19: 2.869(8) Å; O25-H25B O35: 2.977(16) Å; O26-H26B O28: 2.738(9) Å; O27-H27A O20: 2.930(9) Å; O27-H27B O22: 2.975(9) Å; O28-H28D O35: 2.734(11) Å; O28-H28E O31: 2.726(11) Å; O31-H31A O29: 2.807(11) Å]. Views of the structure can be seen in Supplementary Figure S5.

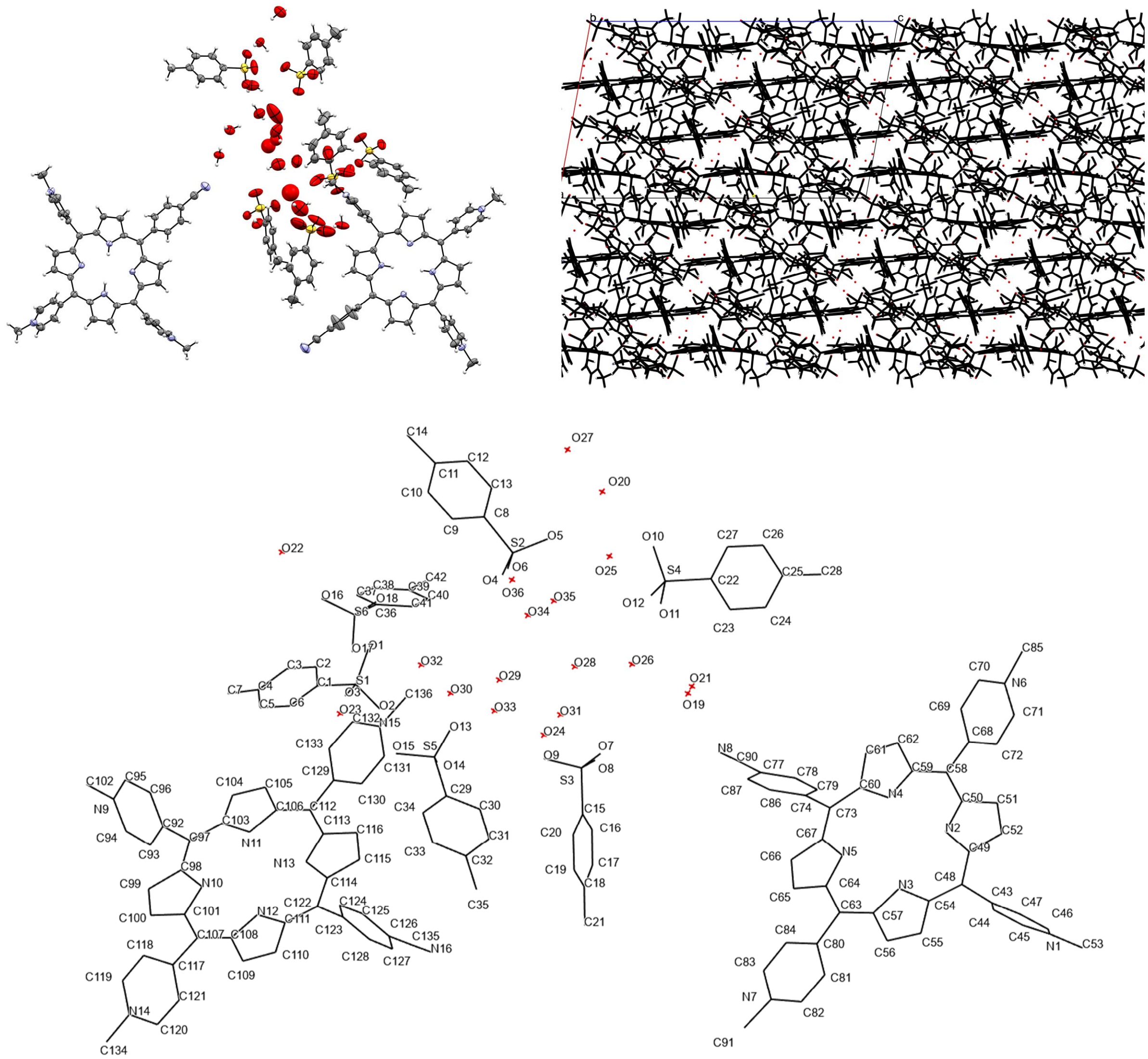

Supplementary Figure S5

Top left: Displacement ellipsoid representation of **[TriMCNP]·[*p*-tosylate]$_3$·(H_2O)$_{8.75}$**. Ellipsoids are drawn at 50% probability. The minor part of a disordered phenylene ring, two disordered hydrogen atoms in the center of one porphyrin ring and a water molecule are omitted for clarity.

Top right: Packing diagram of **[TriMCNP]·[*p*-tosylate]$_3$·(H_2O)$_{8.75}$**, viewed down the [010] direction.

Bottom: Atom-labelling scheme for **[TriMCNP]·[*p*-tosylate]$_3$·(H_2O)$_{8.75}$**. All hydrogen atoms are omitted for clarity.

[TriMCNP]·[*p*-tosylate]$_3$·(H$_2$O)$_7$

Crystalline material was obtained from the gel crystallization of 5,10,15-tris(methylpyridinium)-20-benzonitrile-porphyrin nitrate (**[TriMCNP](NO$_3$)$_3$**), 0.11 M) in agarose (0.5 vol. %) and sodium *p*-toluenesulfonate in water (0.15 M). The gel containing TriMCNP (1.00 mL) was added to a screw cap vial and was covered with agarose gel (1.50 mL). After solidification, sodium *p*-toluenesulfonate (0.15 M, 0.50 mL) was added on top. Tricationic 5,10,15-tris(methylpyridinium)-20-benzonitrile-porphyrin crystallized with *p*-toluenesulfonate counter-ions as a heptahydrate in the monoclinic space group $P2_1/c$. The asymmetric unit consists of two trivalent 5,10,15-tris(methylpyridinium)-20-benzonitrile-porphyrin cations, six *p*-toluenesulfonate counter-ions and 14 water molecules (Z' = 2). The structural formula in the refinement model is $C_{45}H_{35}N_8$, 3($C_7H_7O_3S$), 6.5(H_2O), 0.5(HO) (the location of the second hydrogen atom of the latter water molecule could not be found). Within the porphyrin ring, hydrogen bonds are formed between the nitrogen atoms [N3-H3 N2: 2.889(3) Å; N4-H4 N5: 2.885(3) Å; N10-H10 N11: 2.904(3) Å]. Additionally, the cyanide group of one of the porphyrins accepts a hydrogen bond from a free water molecule [O22-H22B N16: 2.903(3) Å]. The sulfonate groups of the *p*-toluenesulfonate anions also accept hydrogen bonds from free water molecules [O23-H23A O11: 2.834(3) Å; O24-H24B O5: 2.818(3) Å; O27-H27B O17: 2.869(19) Å; O28-H28D O3: 2.761(4) Å; O29-H29A O3: 2.750(4) Å; O33-H33A O7: 2.893(5) Å; O34-H34A O6: 2.781(4) Å; O35-H35D O14: 2.818(7) Å]. Also, the free water molecules form hydrogen bonds among themselves [O22-H22A O24: 2.757(3) Å; O23-H23B O1: 2.916(3) Å; O24-H24A O29: 2.807(4) Å; O25-H25A O13: 2.982(19) Å; O25-H25A O18: 2.774(6) Å; O25-H25B O21: 2.819(3) Å; O26-H26A O20: 2.854(4) Å; O26-H26B O22: 2.863(4) Å; O27-H27A O8: 2.878(4) Å; O29-H29B O34: 2.779(5) Å; O30-H30A O23: 2.925(4) Å; O30-H30B O26: 2.977(4) Å; O31-H31A O29: 2.729(4) Å; O31-H31B O33: 2.753(5) Å; O32-H32A O34: 2.711(5) Å; O33-H33B O32: 2.789(5) Å]. Views of the structure can be seen in Supplementary Figure S6.

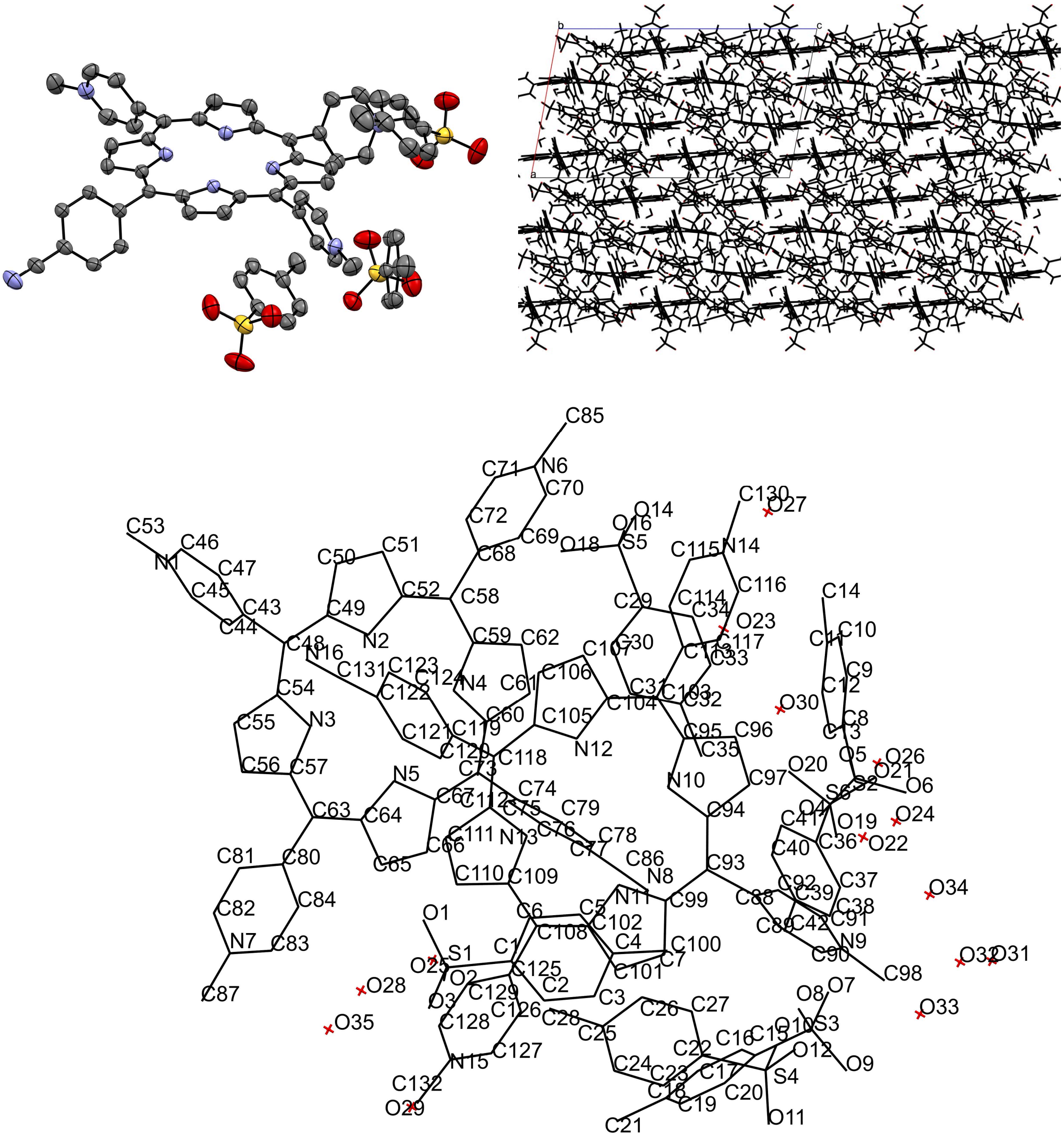

Supplementary Figure S6

Top left: Displacement ellipsoid representation of **[TriMCNP]·[*p*-tosylate]$_3$·(H$_2$O)$_7$**. Ellipsoids are drawn at 50% probability. Only one porphyrin cation out of the two in the asymmetric unit and the corresponding three tosylate anions are shown. All hydrogen atoms, the minor part of a disordered phenylene ring and the water molecules are omitted for clarity.

Top right: Packing diagram of **[TriMCNP]·[*p*-tosylate]$_3$·(H$_2$O)$_7$**, viewed down the [010] direction.

Bottom: Atom-labelling scheme for **[TriMCNP]·[*p*-tosylate]$_3$·(H$_2$O)$_7$**. All hydrogen atoms are omitted for clarity.

[TriMCOOP][besylate]$_3$·(H_2O)$_4$

Crystalline material was obtained from vapor diffusion crystallization. Triscationic 5-(4'-carboxyphenyl)-10,15-20-tri(N-methyl-4'-pyridinium porphyrin crystallized with benzenesulfonate anions as a tetrahydrate in the monoclinic space group $P2_1/c$. The asymmetric unit comprises one trivalent 5-(4'-carboxyphenyl)-10,15-20-tri(*N*-methyl-4'-pyridinium)porphyrin cation, three benzenesulfonate anions and four water molecules, one of which is disordered (occupancy ratio of the disordered sites: 62:38). The structural formula is $C_{45}H_{36}N_7O_2$, 3($C_6H_5O_3S$), 4(H_2O). The carboxylic acid group bound to the porphyrin forms a hydrogen bond with each position of the disordered water molecule [O1-H1 O21: 2.506(5) Å; O1-H1 O21B: 2.669(8) Å]. The sulfonate groups of the benzenesulfonate anions accept hydrogen bonds from free water molecules [O21-H21A O13: 2.746(6) Å; O21B-H21A O13B: 2.684(10) Å; O21-H21B O14: 2.727(6) Å; O21B-H21B O14: 2.701(7) Å; O22-H22A O15: 2.857(6) Å; O25-H25A O19: 2.809(4) Å; O26-H26A O15: 2.870(6) Å; O26-H26B O17: 2.883(5) Å; O25-H25B O12B: 2.792(9)]. Additionally, the free water molecules form hydrogen bonds among themselves [O22-H22B O25: 2.795(5) Å]. Views of the structure can be seen in Supplementary Figure S7.

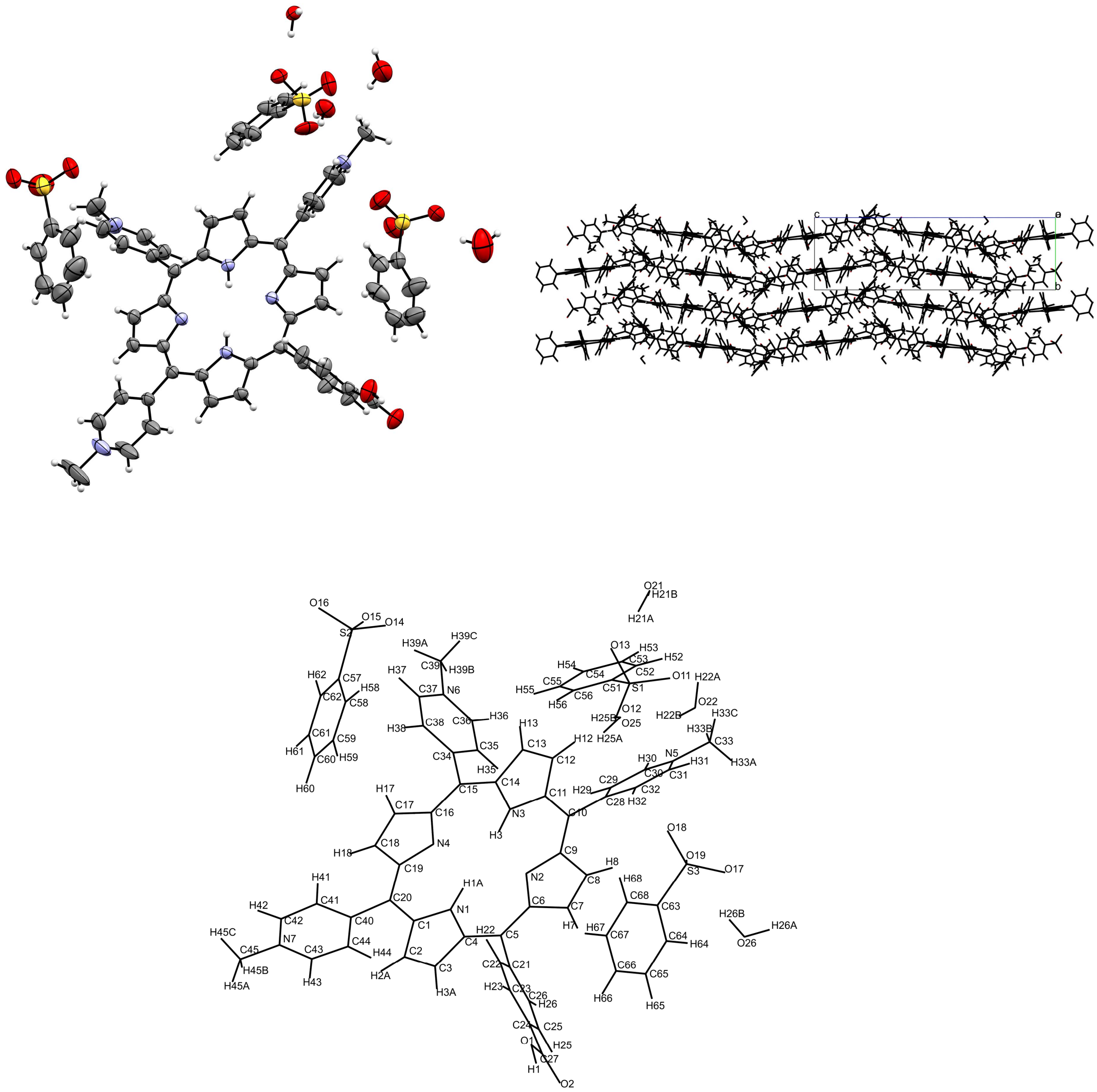

Supplementary Figure S7

Top left: Displacement ellipsoid representation of **[TriMCOOP][besylate]$_3$·(H$_2$O)$_4$**. Ellipsoids are drawn at 50% probability. The minor part of a disordered phenylene ring is omitted for clarity.

Top right: Packing diagram of **[TriMCOOP][besylate]$_3$·(H$_2$O)$_4$**, viewed down the [100] direction.

Bottom: Atom-labelling scheme for **[TriMCOOP][besylate]$_3$·(H$_2$O)$_4$**.

Crystallographic Data

Supplementary Table S4 Crystallographic Data of the **[TMPyP]$^{4+}$** salts.

name	**[TMPyP]Br_4 ·$(H_2O)_6$**	**[TMPyP] ·[nitrate]$_4$ ·$(H_2O)_{2.5}$**	**[TMPyP] ·[1-naphthalene sulfonate]$_4$**	**[TMPyP] ·[2,6-naphthalene disulfonate]$_2$ ·$(H_2O)_{7.5}$**
Squeeze (Spek, 2015) used	no	yes	yes	no
CCDC number	2465396	2465394	2465395	2465393
Empirical formula	$C_{44}H_{50}Br_4N_8O_6$	$C_{44}H_{38}N_{12}O_{14.50}$	$C_{84}H_{68}N_8O_{14}S_4$	$C_{64}H_{66}N_8O_{20.5}S_4$
Formula weight	1106.56	966.86	1541.70	1403.48
Crystal system	Triclinic	Triclinic	Triclinic	Triclinic
Space group	$P\bar{1}$	$P\bar{1}$	$P\bar{1}$	P-1
a [Å]	10.15908(14)	6.38190(9)	15.19786(17)	14.7794(2)
b [Å]	14.97284(18)	16.2135(2)	17.1894(2)	15.2582(2)
c [Å]	15.76491(13)	25.1193(3)	17.9323(2)	16.0581(3)
α [°]	81.1416(8)	82.8804(11)	116.0657(13)	72.3910(10)
β [°]	76.1519(9)	87.3709(11)	94.3459(11)	68.206(2)
γ [°]	88.1501(11)	81.8943(11)	106.3405(11)	89.5920(10)
Volume [$Å^3$]	2300.51(5)	2552.29(6)	3928.68(10)	3182.12(10)
Z	2	2	2	2
Density (calculated) [Mg/m^3]	1.597	1.258	1.303	1.465
Temperature [K]	159.99(10)	159.98(10)	159.99(10)	160.01(19)
Wavelength [Å]	1.54184	1.54184	1.54184	1.54184
Absorption coefficient [mm^{-1}]	4.729	0.820	1.686	2.092
F(000)	1116	1004	1608	1468
Crystal size [mm^3]	0.146 x 0.058 x 0.04	0.211 x 0.051 x 0.035	0.101 x 0.062 x 0.025	0.24 x 0.092 x 0.055
Crystal	red needle	red needle	red plate	red needle

description				
θ range [°]	2.920 to 79.214	2.773 to 79.344	2.820 to 79.489	3.060 to 79.643
Index ranges	-12<=h<=12, -18<=k<=19, -13<=l<=19	-7<=h<=8, -18<=k<=20, -31<=l<=31	-19<=h<=19, -21<=k<=13, -20<=l<=22	-18<=h<=18, -19<=k<=16, -20<=l<=18
Reflections collected	45971	53886	61206	89349
Indep. reflections	9864 [R(int) = 0.0354]	10919 [R(int) = 0.0429]	16109 [R(int) = 0.0328]	13577 [R(int) = 0.0385]
Reflections observed	8634	9365	12127	12530
Criterion for observation	I > 2 σ (I)	I > 2 σ (I)	I > 2 σ (I)	I > 2 s (I)
Completeness to θ	99.9% to 67.684°	99.9% to 67.684°	99.6% to 67.684°	99.8 % to 67.684°
Absorption correction	Analytical	Semi-empirical from equival.	Gaussian	Gaussian
Max. and Min. transm.	0.852 and 0.624	1.00000 and 0.83418	1.000 and 0.860	1.000 and 0.554
Data/ restraints/ parameters	9864 / 4 / 590	10919 / 37 / 647	16109 / 47 / 1141	13577 / 30 / 938
Goodness-of-fit on F^2	1.094	1.027	1.050	1.161
Final R indices [I > 2 σ (I)]	R1 = 0.0382, wR2 = 0.1064	R1 = 0.1019, wR2 = 0.3085	R1 = 0.0753, wR2 = 0.2097	R1 = 0.1021, wR2 = 0.3257
R indices (all data)	R1 = 0.0435, wR2 = 0.1100	R1 = 0.1090, wR2 = 0.3177	R1 = 0.0946, wR2 = 0.2275	R1 = 0.1074, wR2 = 0.3292
Largest diff. peak and hole [e.Å^{-3}]	0.698 and -0.658	1.144 and -0.528	1.252 and -0.639	0.949 and -0.543

Supplementary Table S5 Crystallographic data of the **[TriMCNP]$^{3+}$** salts.

name	**[TriMCNP][*p*-tosylate]$_3$ ·$(H_2O)_{8.75}$** (vapor diffusion)	**[TriMCNP][*p*-tosylate]$_3$ ·$(H_2O)_7$** (gel crystallization)

Squeeze (Spek, 2015) used	no	yes
CCDC number	2465391	2465392
Empirical formula	$C_{66}H_{79}N_8O_{17.75}S_3$	$C_{66}H_{69.5}N_8O_{16}S_3$
Formula weight	1364.55	1326.97
Crystal system	Monoclinic	Monoclinic
Space group	$P2_1/c$	$P2_1/c$
a [Å]	17.50074(18)	17.54006(8)
b [Å]	25.9751(3)	25.99854(12)
c [Å]	30.1002(4)	30.05455(13)
α [°]	90	90
β [°]	100.4228(12)	100.5017(4)
γ [°]	90	90
Volume [$Å^3$]	13457.2(3)	13475.78(10)
Z	8	8
Density (calculated) [Mg/m^3]	1.347	1.308
Temperature [K]	159.99(10)	160.15
Wavelength [Å]	1.54184	1.54184
Absorption coefficient [mm^{-1}]	1.645	1.611
F(000)	5768	5580
Crystal size [mm^3]	0.44 x 0.034 x 0.03	0.267 x 0.153 x 0.142
Crystal description	red needle	red needle
θ range [°]	2.263 to 47.692	2.263 to 79.242
Index ranges	-16<=h<=13, -24<=k<=24, -28<=l<=28	-21<=h<=22, -32<=k<=31, -38<=l<=37
Reflections collected	99768	275353
Indep. reflections	12390 [R(int) = 0.0470]	28905 [R(int) = 0.0389]
Reflections observed	10103	25118
Criterion for observation	I > 2 σ (I)	I > 2 s (I)
Completeness to θ	99.7% to 47.692°	100.0 % to 67.684°
Absorption correction	Gaussian	Gaussian
Max. and Min. transm.	1.000 and 0.915	1.000 and 0.416
Data/ restraints/ parameters	12390 / 13 / 1812	28905 / 230 / 1761

Goodness-of-fit on F^2	1.032	1.057
Final R indices [I > 2 σ (I)]	R1 = 0.0777, wR2 = 0.2123	R1 = 0.0716, wR2 = 0.2109
R indices (all data)	R1 = 0.0929, wR2 = 0.2275	R1 = 0.0780, wR2 = 0.2184
Largest diff. peak and hole [e.Å^{-3}]	1.619 and -0.591	1.819 and -1.357

Supplementary Table S6. Crystallographic data of **[TriMCOOP][besylate]$_3$·(H$_2$O)$_4$**.

Name	**[TriMCOOP][besylate]$_3$·(H$_2$O)$_4$**
Squeeze (Spek, 2015) used	yes
CCDC number	2465390
Empirical formula	$C_{63}H_{59}N_7O_{15}S_3$
Formula weight	1250.35
Crystal system	Monoclinic
Space group	$P2_1/n$
a [Å]	20.92834(15)
b [Å]	9.39850(8)
c [Å]	32.2135(2)
a [°]	90
b [°]	102.9338(7)
g [°]	90
Volume [Å3]	6175.48(8)
Z	4
Density (calculated) [Mg/m3]	1.345
Temperature [K]	159.99(10)
Wavelength [Å]	1.54184
Absorption coefficient [mm-1]	1.709
F(000)	2616
Crystal size [mm3]	0.175 x 0.091 x 0.046
Crystal description	red plate
Theta range for data collection [°]	4.295 to 78.885
Index ranges	-24<=h<=26, -11<=k<=11, -40<=l<=40
Reflections collected	87383
Independent reflections	13115 [R(int) = 0.0418]
Reflections observed	11510
Criterion for observation	I > 2 s (I)
Completeness to theta	99.8 % to 67.684°
Absorption correction	Gaussian
Max. and min. transmission	1.000 and 0.571
Data / restraints / parameters	13115 / 54 / 863
Goodness-of-fit on F2	1.064
Final R indices [I > 2 s (I)]	R1 = 0.0730, wR2 = 0.1952
R indices (all data)	R1 = 0.0798, wR2 = 0.1999

Largest diff. peak and hole [e.Å-3]	0.797 and -0.659

Pictures of Crystallization Experiments in Crystal Farm

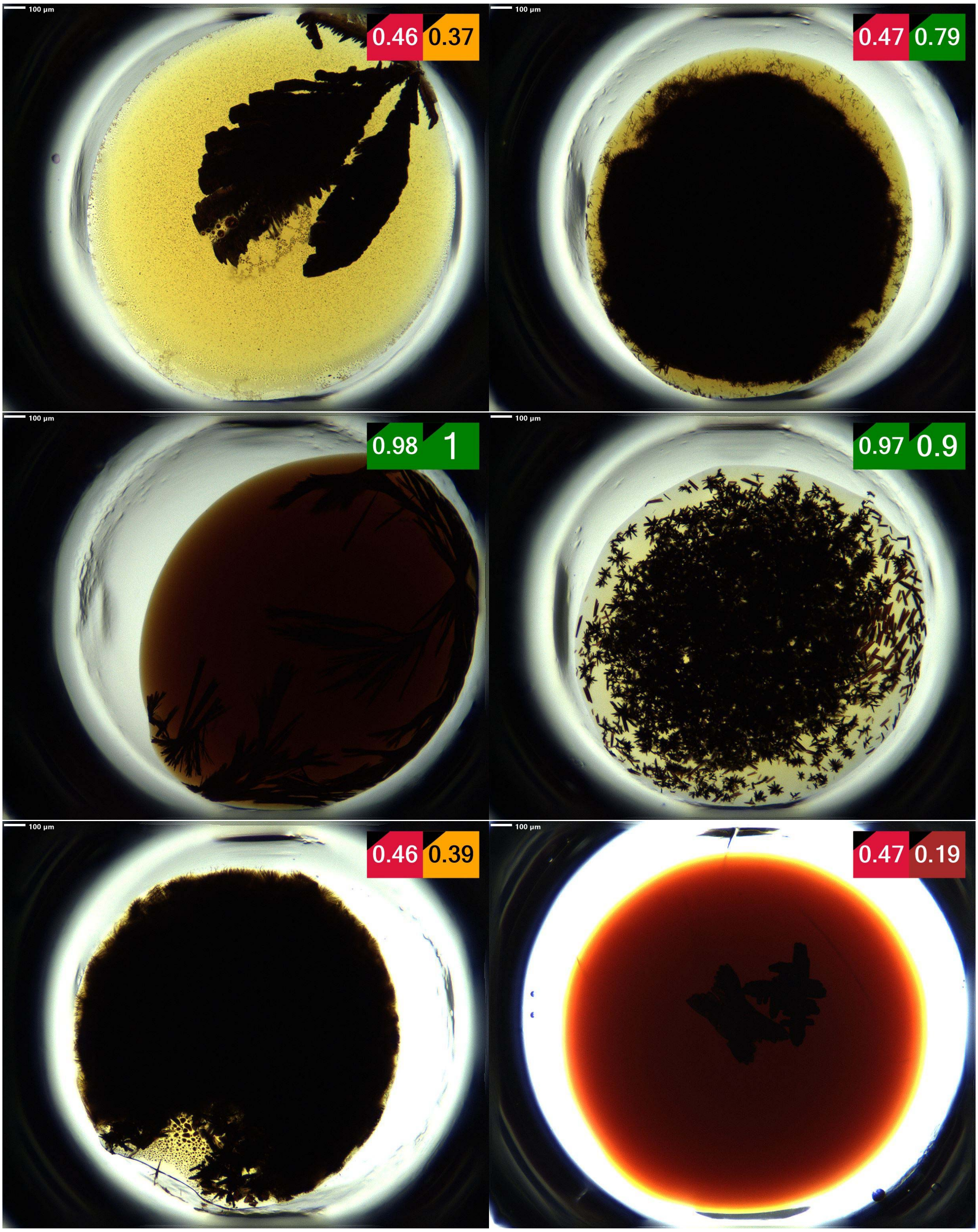

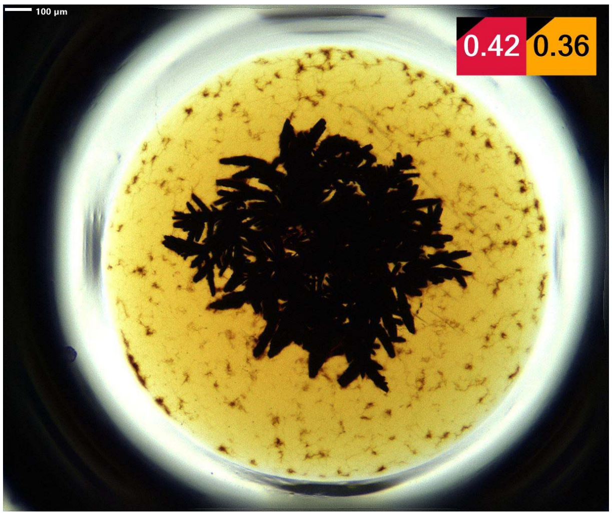

Supplementary Figure S8 Pictures of the results of high-throughput crystallization experiments in the crystal farm after 10 days. Shown are crystallizations using **[TMPyP]Cl_4** (from left to right).

1st row: E4 (0.33 M sodium diphenylacetate), G8 (0.26 M disodium (+)-O,O'-dibenzoyl-D-tartrate);

2nd row: H9 (4 M sodium dihydrogen phosphate), A3 (4 M NaBr);

3rd row: C3 (0.35 M sodium 1-naphthalensulfonate), C5 (0.085 M disodium 2,6-naphthalenedisulfonate);

4th row: C6 (4.6 M sodium nitrate).

Summary of the NSD (in Å) of **[TMPyP]Br_4·$(H_2O)_6$**:

basis	Δ_{ip}	δ_{ip}	B_{2g}	B_{1g}	$E_u(x)$	$E_u(y)$	A_{1g}	A_{2g}
min.	0.25	0.00	-0.05	0.11	-0.01	0.00	0.22	0.01
ext.	0.25	0.00	-0.05	0.11	-0.01	-0.01	0.22	0.01
			-0.02	-0.01	-0.01	-0.01	-0.02	0.00
total	0.26	0.00	-0.05	0.11	-0.01	0.00	0.22	0.01
			-0.02	-0.01	-0.01	-0.01	-0.02	0.00
			0.00	-0.01	0.00	0.00	0.04	0.00
			0.00	0.00	0.00	0.01	-0.01	0.00
			0.00	0.00	0.01	0.01	0.01	0.00
			-0.01	0.00	0.00	0.00	0.01	
					0.00	0.00		
					0.00	0.00		
					0.00	0.00		
					0.00	0.00		
					0.00	0.00		
comp.	0.26	0.00	0.05	0.11	0.02	0.02	0.22	0.01

basis	Δ_{oop}	δ_{oop}	B_{2u}	B_{1u}	A_{2u}	$E_g(x)$	$E_g(y)$	A_{1u}
min.	0.40	0.00	0.24	0.05	0.04	-0.26	-0.17	-0.01
ext.	0.42	0.00	0.24	0.05	0.03	-0.26	-0.17	-0.01
			-0.03	0.00	-0.02	0.03	-0.10	0.00
total	0.42	0.00	0.24	0.05	0.03	-0.26	-0.17	-0.01
			-0.03	0.00	-0.02	0.03	-0.10	0.00
			0.00	0.00	0.00	0.05	-0.01	
						0.03	0.00	
						-0.02	0.00	
comp.	0.42	0.00	0.24	0.05	0.04	0.27	0.20	0.01

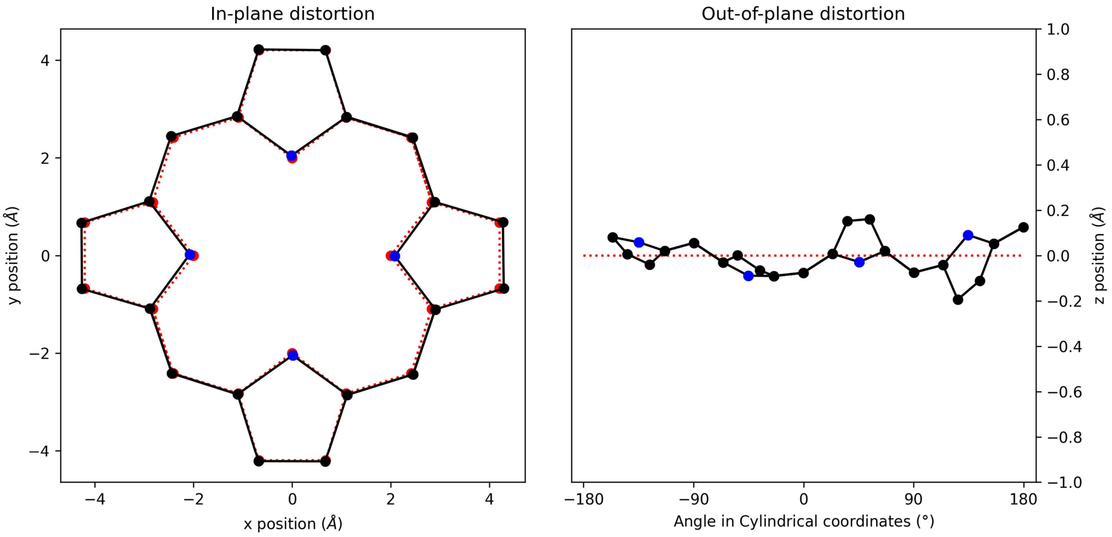

Supplementary Figure S9 (a) out-of-plane and (b) in-plane skeletal plots of the **$[TMPyP]^{4+}$** porphyrin core. The porphyrin atoms are represented in black (C) and blue (N), with the reference structure (CuTPP) shown as red dotted lines.

Summary of the NSD (in Å) of **[TMPyP][nitrate]$_4$·(H$_2$O)$_{2.5}$** first molecule:

basis	Δ_{ip}	δ_{ip}	B_{2g}	B_{1g}	$E_u(x)$	$E_u(y)$	A_{1g}	A_{2g}
min.	0.23	0.00	-0.05	0.08	0.00	0.00	0.21	-0.01
ext.	0.24	0.00	-0.05	0.08	0.00	0.00	0.21	-0.01
			-0.03	0.07	0.00	0.00	-0.01	0.00
total	0.26	0.00	-0.05	0.09	0.00	0.00	0.21	-0.01
			-0.03	0.07	0.00	0.00	-0.01	0.00
			0.00	0.06	0.00	0.00	0.04	0.00
			0.01	0.00	0.00	0.00	0.00	0.01
			0.00	0.00	0.00	0.00	0.01	0.00
			0.00	0.02	0.00	0.00	0.01	
					0.00	0.00		
					0.00	0.00		
					0.00	0.00		
					0.00	0.00		
					0.00	0.00		
comp.	0.26	0.00	0.06	0.12	0.00	0.00	0.22	0.01

basis	Δ_{oop}	δ_{oop}	B_{2u}	B_{1u}	A_{2u}	$E_g(x)$	$E_g(y)$	A_{1u}
min.	0.20	0.01	0.00	0.00	0.00	0.14	0.15	0.00
ext.	0.28	0.00	0.00	0.00	0.00	0.14	0.14	0.00
			0.00	0.00	0.00	0.14	-0.14	0.00
total	0.29	0.00	0.00	0.00	0.00	0.14	0.14	0.00
			0.00	0.00	0.00	0.14	-0.14	0.00
			0.00	0.00	0.00	0.01	-0.04	
						-0.01	-0.02	
						-0.01	0.00	
comp.	0.29	0.00	0.00	0.00	0.00	0.20	0.20	0.00

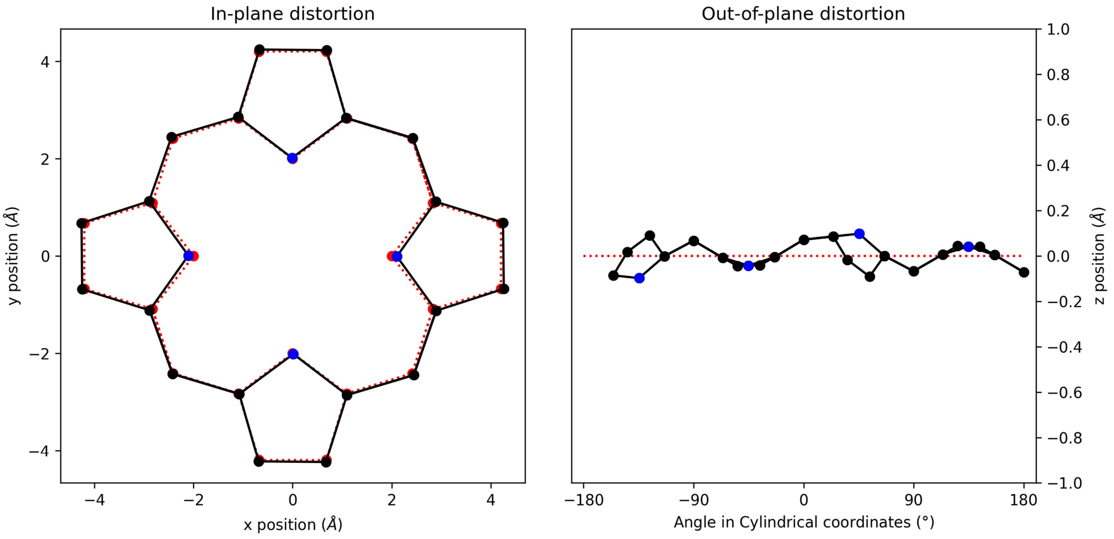

Supplementary Figure S10 (a) out-of-plane and (b) in-plane skeletal plots of the **[TMPyP]$^{4+}$** porphyrin core. The porphyrin atoms are represented in black (C) and blue (N), with the reference structure (CuTPP) shown as red dotted lines.

Summary of the NSD (in Å) of **[TMPyP][nitrate]$_4$·(H$_2$O)$_{2.5}$** second molecule:

basis	Δ_{ip}	δ_{ip}	B_{2g}	B_{1g}	$E_u(x)$	$E_u(y)$	A_{1g}	A_{2g}
min.	0.22	0.00	-0.01	0.02	0.00	0.00	0.22	-0.02
ext.	0.23	0.00	-0.01	0.02	0.00	0.00	0.22	-0.02
			0.00	0.08	0.00	0.00	-0.01	0.00
total	0.25	0.00	-0.01	0.02	0.00	0.00	0.22	-0.02
			0.00	0.08	0.00	0.00	-0.01	0.00
			0.00	0.06	0.00	0.00	0.05	0.00
			0.00	0.00	0.00	0.00	-0.01	0.00
			0.00	-0.01	0.00	0.00	0.01	0.00
			0.00	0.01	0.00	0.00	0.01	
					0.00	0.00		
					0.00	0.00		
					0.00	0.00		
					0.00	0.00		
					0.00	0.00		
comp.	0.25	0.00	0.01	0.10	0.00	0.00	0.23	0.02

basis	Δ_{oop}	δ_{oop}	B_{2u}	B_{1u}	A_{2u}	$E_g(x)$	$E_g(y)$	A_{1u}
min.	0.27	0.00	0.00	0.00	0.00	0.08	-0.25	0.00
ext.	0.30	0.00	0.00	0.00	0.00	0.08	-0.26	0.00
			0.00	0.00	0.00	-0.11	-0.06	0.00
total	0.30	0.00	0.00	0.00	0.00	0.08	-0.25	0.00
			0.00	0.00	0.00	-0.11	-0.06	0.00
			0.00	0.00	0.00	-0.02	0.02	
						-0.01	0.03	
						0.01	0.00	
comp.	0.30	0.00	0.00	0.00	0.00	0.14	0.26	0.00

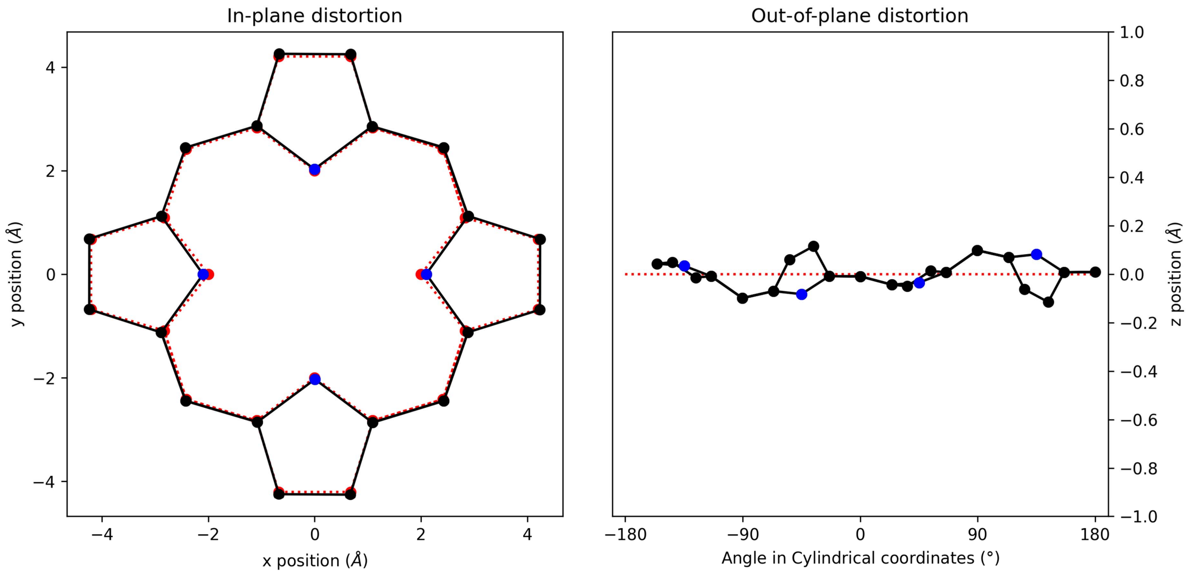

Supplementary Figure S11 (a) out-of-plane and (b) in-plane skeletal plots of the **[TMPyP]$^{4+}$** porphyrin core. The porphyrin atoms are represented in black (C) and blue (N), with the reference structure (CuTPP) shown as red dotted lines.

Summary of the NSD (in Å) of **[TMPyP][1-naphthalenesulfonate]$_4$** first molecule:

**basis**	**Δ_{ip}**	**δ_{ip}**	**B_{2g}**	**B_{1g}**	**$E_u(x)$**	**$E_u(y)$**	**A_{1g}**	**A_{2g}**
min.	0.21	0.00	-0.05	-0.01	0.00	0.00	0.21	-0.02
ext.	0.22	0.00	-0.05	-0.01	0.00	0.00	0.21	-0.02
			-0.01	-0.05	0.00	0.00	-0.01	0.00
total	0.23	0.00	-0.05	-0.01	0.00	0.00	0.21	-0.02
			-0.01	-0.05	0.00	0.00	-0.01	0.00
			0.00	-0.03	0.00	0.00	0.05	0.00
			0.00	0.00	0.00	0.00	-0.01	0.00
			-0.01	0.00	0.00	0.00	0.01	0.00
			0.00	-0.02	0.00	0.00	0.01	
					0.00	0.00		
					0.00	0.00		
					0.00	0.00		
					0.00	0.00		
					0.00	0.00		
comp.	0.23	0.00	0.05	0.07	0.00	0.00	0.22	0.02

**basis**	**Δ_{oop}**	**δ_{oop}**	**B_{2u}**	**B_{1u}**	**A_{2u}**	**$E_g(x)$**	**$E_g(y)$**	**A_{1u}**
min.	0.16	0.00	0.00	0.00	0.00	0.02	-0.16	0.00
ext.	0.21	0.00	0.00	0.00	0.00	0.02	-0.16	0.00
			0.00	0.00	0.00	-0.07	-0.11	0.00
total	0.21	0.00	0.00	0.00	0.00	0.02	-0.16	0.00
			0.00	0.00	0.00	-0.07	-0.11	0.00
			0.00	0.00	0.00	-0.01	0.00	
						-0.01	0.02	
						0.00	-0.01	
comp.	0.21	0.00	0.00	0.00	0.00	0.07	0.20	0.00

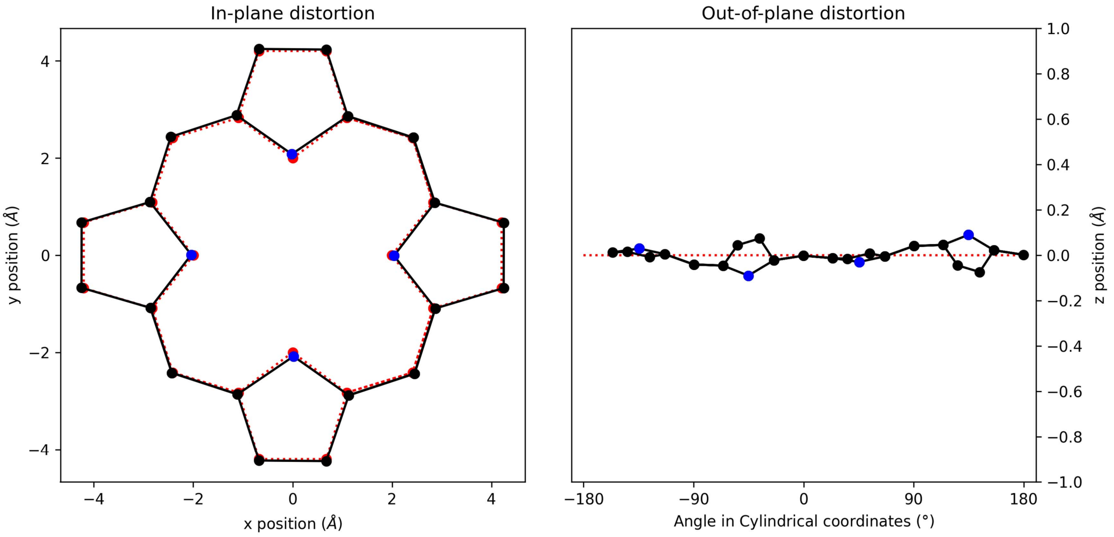

Supplementary Figure S12 (a) out-of-plane and (b) in-plane skeletal plots of the **[TMPyP]$^{4+}$** porphyrin core. The porphyrin atoms are represented in black (C) and blue (N), with the reference structure (CuTPP) shown as red dotted lines.

Summary of the NSD (in Å) of **[TMPyP][1-naphthalenesulfonate]$_4$** second molecule:

basis	Δ_{ip}	δ_{ip}	B_{2g}	B_{1g}	$E_u(x)$	$E_u(y)$	A_{1g}	A_{2g}
min.	0.23	0.00	0.01	-0.09	0.00	0.00	0.21	0.00
ext.	0.25	0.00	0.01	-0.09	0.00	0.00	0.21	0.00
			-0.01	-0.08	0.00	0.00	-0.01	0.00
total	0.27	0.00	0.01	-0.10	0.00	0.00	0.22	0.00
			-0.01	-0.09	0.00	0.00	-0.01	0.00
			0.00	-0.07	0.00	0.00	0.05	0.00
			0.00	0.00	0.00	0.00	-0.01	0.00
			0.00	0.00	0.00	0.00	0.01	0.00
			0.00	-0.02	0.00	0.00	0.01	
					0.00	0.00		
					0.00	0.00		
					0.00	0.00		
					0.00	0.00		
					0.00	0.00		
comp.	0.27	0.00	0.01	0.15	0.00	0.00	0.22	0.01

basis	Δ_{oop}	δ_{oop}	B_{2u}	B_{1u}	A_{2u}	$E_g(x)$	$E_g(y)$	A_{1u}
min.	0.11	0.00	0.00	0.00	0.00	0.00	-0.11	0.00
ext.	0.13	0.00	0.00	0.00	0.00	0.01	-0.11	0.00
			0.00	0.00	0.00	0.07	-0.02	0.00
total	0.13	0.00	0.00	0.00	0.00	0.01	-0.11	0.00
			0.00	0.00	0.00	0.07	-0.02	0.00
			0.00	0.00	0.00	0.02	0.00	
						0.01	0.02	
						0.00	0.00	
comp.	0.13	0.00	0.00	0.00	0.00	0.08	0.11	0.00

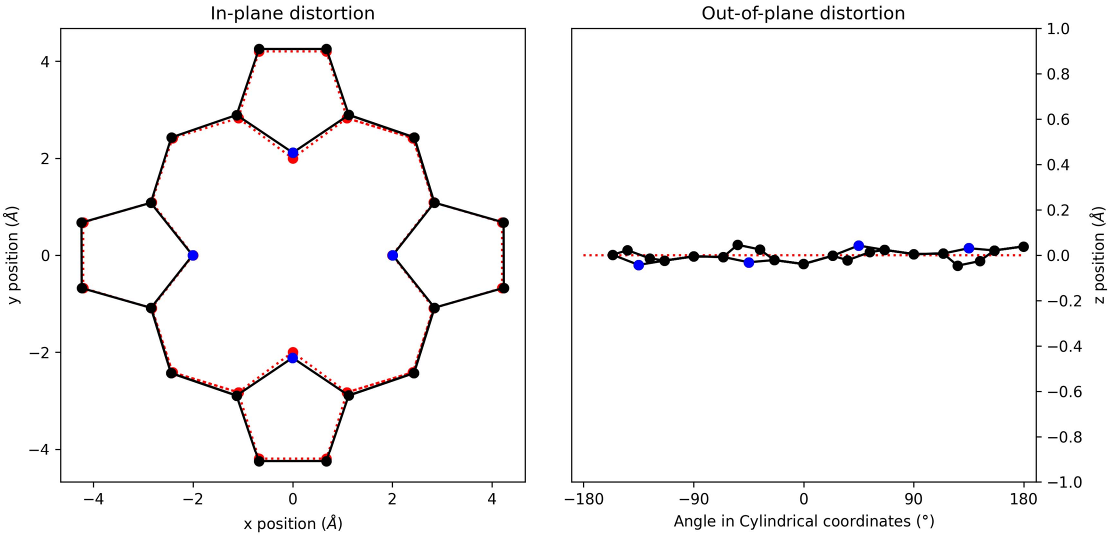

Supplementary Figure S13 (a) out-of-plane and (b) in-plane skeletal plots of the **[TMPyP]$^{4+}$** porphyrin core. The porphyrin atoms are represented in black (C) and blue (N), with the reference structure (CuTPP) shown as red dotted lines.

Summary of the NSD (in Å) of **[TMPyP][2,6-naphthalenedisulfonate]$_2$·(H$_2$O)$_{7.5}$**:

**basis**	Δ_{ip}	δ_{ip}	B_{2g}	B_{1g}	$E_u(x)$	$E_u(y)$	A_{1g}	A_{2g}
min.	0.09	0.01	0.05	-0.07	0.00	0.02	0.00	0.00
ext.	0.14	0.01	0.05	-0.07	0.00	0.02	0.00	0.00
			0.01	-0.07	0.00	0.00	-0.09	0.01
total	0.21	0.00	0.05	-0.07	0.00	0.02	0.01	0.00
			0.01	-0.07	0.00	0.00	-0.09	0.01
			0.00	-0.04	0.00	0.01	0.15	0.01
			0.00	0.00	0.00	-0.01	-0.02	0.00
			0.00	0.00	-0.01	-0.02	0.01	0.01
			0.01	0.00	0.00	0.00	0.00	
					0.01	0.00		
					0.01	0.00		
					-0.01	0.00		
					0.01	0.00		
					0.00	0.00		
comp.	0.21	0.00	0.05	0.11	0.02	0.03	0.17	0.01

**basis**	Δ_{oop}	δ_{oop}	B_{2u}	B_{1u}	A_{2u}	$E_g(x)$	$E_g(y)$	A_{1u}
min.	1.77	0.02	1.77	0.06	0.04	0.08	0.02	0.01
ext.	1.79	0.00	1.77	0.06	0.04	0.08	0.02	0.01
			-0.26	-0.01	-0.04	0.01	0.00	0.00
total	1.79	0.00	1.77	0.06	0.04	0.08	0.02	0.01
			-0.26	-0.01	-0.04	0.01	0.00	0.00
			-0.01	0.00	0.01	-0.01	0.01	
						-0.01	0.01	
						0.00	0.00	
comp.	1.79	0.00	1.78	0.06	0.06	0.08	0.02	0.01

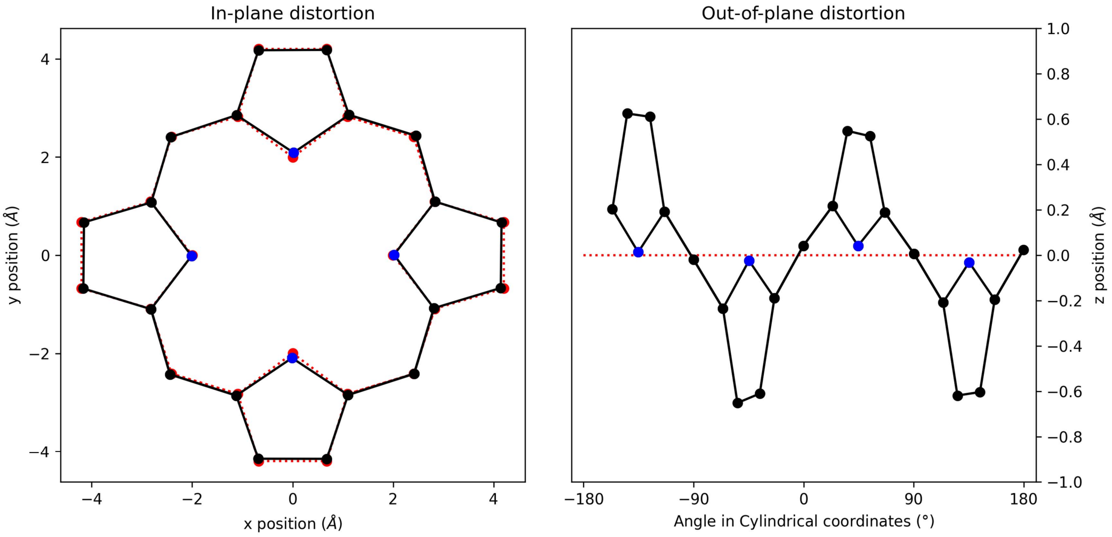

Supplementary Figure S14 (a) out-of-plane and (b) in-plane skeletal plots of the **[TMPyP]$^{4+}$** porphyrin core. The porphyrin atoms are represented in black (C) and blue (N), with the reference structure (CuTPP) shown as red dotted lines.

References

Dolomanov, O. V., Bourhis, L. J., Gildea, R. J., Howard, J. A. K. & Puschmann, H. (2009). *J. Appl. Cryst.* **42**, 339-341.

Macrae, C. F., Sovago, L., Cottrell, S. J., Galek, P. T. A., McCabe, P., Pidcock, E., Platings, M., Shields, G. P., Stevens, J. S., Towler, M. & Wood, P. A. (2020). *J. Appl. Cryst.* **53**, 226-235.

Rigaku Oxford Diffraction (2024). *CrysAlis^{Pro} Software system.* Version 1.171.43. Rigaku Corporation,

Sheldrick, G. M. (2015a). *Acta Cryst.* **A71**, 3-8.

Sheldrick, G. M. (2015b). *Acta Cryst.* **C71**, 3-8.

Spek, A. L. (2015). *Acta Cryst.* **C71**, 9-18.

Spek, A. L. (2023). *Comprehensive Inorganic Chemistry III*, 3rd ed., edited by J. Reedijk & K. R. Poeppelmeier, pp. 425-444. Oxford: Elsevier.

Thorn, A., Dittrich, B. & Sheldrick, G. M. (2012). *Acta Cryst.* **A68**, 448-451.
